# Central Osmolality Sensing for Arginine Vasopressin Release Is Mediated by WNK1‐OSR1/SPAK‐Kv3.1 Cascade

**DOI:** 10.1096/fj.202502072RR

**Published:** 2025-11-17

**Authors:** Xin Jin, Jian Xie, Chia‐Wei Yeh, Yu‐Jui Li, Cheng‐Chang Lien, Chou‐Long Huang

**Affiliations:** ^1^ Department of Medicine, Division of Nephrology University of Iowa Carver College of Medicine Iowa City Iowa USA; ^2^ Institute of Neuroscience National Yang Ming Chiao Tung University Taipei Taiwan; ^3^ Brain Research Center National Yang Ming Chiao Tung University Taipei Taiwan

## Abstract

Life depends on maintaining water homeostasis and internal osmolality constancy. In terrestrial animals, the release of the antidiuretic hormone arginine vasopressin (AVP) in response to variations of extracellular osmolality (tonicity) is crucial. We have reported that WNK1 kinase in the vascular‐organ‐of‐lamina‐terminalis (OVLT) nuclei of the brain mediates the hypertonicity‐induced AVP release by activating the voltage‐gated K^+^ channel Kv3.1 increasing action potential firing. The downstream mechanism for WNK1‐mediated osmosensation is unknown. Here, we showed that the hypertonicity‐induced increases in Kv3.1 currents in cultured cells required the oxidative stress‐responsive‐1 (OSR1) or STE20/SPS1‐related proline/alanine‐rich (SPAK) kinase. Both kinases were present in the mouse OVLT area. Hypertonicity induced by water restriction or mannitol injection increased the abundance of phosphorylated OSR1 and SPAK in the OVLT. Double deletion of *Osr1* and *Spak* in the OVLT in mice caused polyuria with relative hypotonic urine that persisted in water restriction. The water restriction‐induced AVP release was blunted in *Osr1* and *Spak*‐deleted mice. In brain slice recordings, the hypertonicity‐induced increases in action potential firing in OVLT were blunted by *Osr1* and *Spak* deletion. Deletion of the Kv3.1 channel in the OVLT showed a similar phenotype. Expression of the constitutively active OSR1 in the OVLT resulted in increased AVP release and inappropriate antidiuretic hormone secretion phenotype. In summary, OSR1/SPAK acts downstream of WNK1 to regulate AVP release in response to hypertonicity. In OVLT neurons OSR1/SPAK activates Kv3.1 to increase action potential firing. Thus, the WNK1‐OSR1/SPAK‐Kv3.1 cascade regulates water homeostasis and AVP release to control osmolality stability.

## Graphical abstract

Extracellular hyperosmolality extracts water from the catalytic center of WNK1 increasing the kinase activity. WNK1 phosphorylates and activates OSR1/SPAK leading to increases in Kv3.1 activity. Enhanced Kv3.1 activity accelerates repolarization, increasing afterhyperpolarization (AHP) and/or shortening AP width to increase action potential firing.

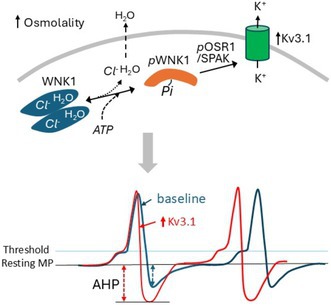

## 1 Introduction

Maintaining osmolality (tonicity) constancy is essential for the integrity of the cell membrane and cellular function. In terrestrial animals, the release of the antidiuretic hormone arginine vasopressin (AVP) plays a crucial role. AVP stimulates water reabsorption in the kidney to regulate total body water homeostasis in response to an increase in the extracellular tonicity. AVP is synthesized by the magnocellular neurosecretory neurons in the paraventricular (PVN) and supraoptic (SON) nuclei in the hypothalamus and transported to the posterior pituitary gland for release into the blood circulation. The organum vasculosum of the lamina terminalis (OVLT) and subfornical organ (SFO), known as circumventricular organs (CVOs), lack a blood–brain barrier positioned to detect changes in serum osmolality. OVLT and SFO neurons detect and relay the signals to the PVN and SON in the form of action potentials (APs).

The molecular identity of the osmolality sensor(s) in the CVOs remains elusive. Membrane resident mechanosensitive channels have been implicated [1, 2, 3]. WNKs (with‐no‐lysine [K]'s) are a group of intracellular protein kinases consisting of four members, WNK1‐4 [4, 5, 6, 7]. WNK1 is ubiquitously expressed including in the brain [6, 7]. Structural studies reveal that the catalytic cavity of the WNK1 kinase domain contains numerous bound water molecules. Water binding inhibits whereas unbinding increases the kinase activity [8]. We have recently reported that WNK1 plays an important role in the hypertonicity‐induced release of AVP [9]. We found that hypertonicity induces WNK1 activation leading to increased activity of the voltage‐gated K^+^ channel Kv3.1 and increases in AP firing [9]. WNKs regulate ion channels and transporters including many types of K^+^ channels, epithelial Na^+^ channel ENaC, cation‐chloride co‐transporters NKCC1, 2, NCC, and KCC [10, 11, 12, 13]. The regulatory mechanisms may be dependent or independent on the kinase activity [12]. The kinase‐dependent mechanisms may involve direct phosphorylation by WNKs or indirectly through other protein kinases including PI4K, MAPK and oxidative‐stress responsive‐1 kinase (OSR1) and related SPAK (Ste20‐related proline/alanine‐rich kinase) [14, 15, 16].

In this study, we report that WNK1 functions as a central osmolality sensor for AVP release by phosphorylating and activating OSR1/SPAK kinase. We show that activation of OSR1/SPAK is essential for hypertonicity stimulation of KV3.1 channel activity. Loss of function of OSR1/SPAK in brain leads to defects in hypertonicity‐induced AVP release and in water homeostasis. Gain‐of‐function of OSR1 in mice enhances AVP release in the absence of hypertonicity stimulation recapitulating the phenotype of patients with syndrome of inappropriate ADH release (SIADH).

## 2 Materials and Methods

### 2.1 Animals

All mice were housed in a temperature‐controlled room with a 12‐h dark/light cycle, with food and water available ad libitum. *Spak* ^*f/f*^, *Osr1* ^*f/f*^, and *CA‐OSR1* ^*f/f*^ mice have been described and genotyped before [17, 18]. *Kcnc1* ^*f/f*^ mice were from Shanghai Model Organisms. Briefly, these mice carry two loxP sites flanking the target exon 2 of the *Kcnc1* gene; Cre recombinase excision of the allele results in exon 2 excision, which eliminates *Kcnc1* coding in specific tissues. Male mice with ages between 3 and 5 months were used for the electrophysiological experiments. Age‐matched adult male mice were used in metabolic cage experiments.

### 2.2 Cell Line and Reagents

Established human embryonic kidney 293 (HEK293) cells were obtained from the American Type Culture Collection (ATCC CRL‐1573) and cultured at 37°C in Dulbecco's modified Eagle's medium (DMEM) supplemented with 10% fetal bovine serum (FBS) and 1% penicillin/streptomycin (all from ATCC) in a humidified atmosphere of 5% CO_2_/95% air. The retrograde AAVrg‐cre virus was from Addgene (catalog 24593‐AAVrg and 55632‐AAVrg) [19, 20]. AAV2/5‐Cre‐EGFP was from the Viral Vector Core, University of Iowa. The following primary and secondary antibodies were used: anti‐SPAK (2281, Cell Signaling); anti‐OSR (3729, Cell Signaling); GFP antibody (MA5‐15256, Invitrogen); anti‐Kv3.1 (NBP2‐12903, Novus Biologicals); anti‐GAPDH‐HRP (sc‐47724 HRP, Santa Cruz). Alexa Fluor secondary antibody (A‐11012) was from Thermo Fisher Scientific. Anti‐rabbit IgG‐HRP (4030–05, Southernbiotech), anti‐mouse IgG‐HRP (1030–05, Southernbiotech) were used. Anti‐phospho‐SPAK/OSR1 antibody (07–2273), TEA, was obtained from Sigma‐Aldrich.

### 2.3 Immunostaining

Mice were euthanized and perfused with cold PBS and 4% PFA. The brains were dissected and fixed in 2% PFA overnight, processed through 10% and 30% sucrose, embedded with OCT, and frozen. Frozen sections were cut coronally at 25–30 μm thickness. Brain sections were blocked by 4% normal goat serum and 1% BSA in 0.3% Tris‐Triton solution for 2 h and then incubated with the primary antibody in blocking buffer overnight at 4°C. The sections were washed with PBS containing 0.3% Tris‐Triton 3 times and then incubated with second antibodies at room temperature for 1.5 h. The slides were then washed, mounted, and examined with an Olympus BX61 microscope.

### 2.4 Western Blotting

RIPA buffer containing a protease and phosphatase inhibitor cocktail (Sigma‐Aldrich) was used to homogenize the dissected mouse brain tissues (OVLT, cortex, and PVN) while being gently shaken for an hour. The entire lysate was centrifuged for 30 min at 4°C at 15700 ***g***. BCA was used to measure the protein content of the obtained supernatant. Lysate samples (~20 μg protein) were heated for 5 min in 4× SDS‐PAGE sample buffer (4% SDS, 20% glycerol, 10% 2‐mercaptoethanol, 0.004% bromophenol blue, and 0.125 M Tris HCl, pH 6.8) in order to perform Western blotting analysis. They were then separated using precast 4%–12% Bis‐Tris Gels (NuPAGE) and electroblotted onto a PVDF membrane. After an hour of incubation in blocking solution (5% milk + Tween‐20), the membranes were incubated overnight at 4°C with a primary antibody diluted in blocking buffer. Prior to being incubated with a secondary antibody, the membranes were washed in TBS containing 0.1% Tween‐20. Bio‐Rad ECL Substrates were used to identify bound antibodies. Densitometry was used to quantify the bands using ImageJ (NIH).

### 2.5 Metabolic Cage Studies, Urine, and Blood Analysis

Urine and water intake were measured in mouse metabolic cages (Hatteras Instruments). Measurements of water intake and urine volume are averages from two to three studies. The mice were given a stereotaxic injection and given at least 7 days to recuperate before their water and urine intakes were recorded. For water deprivation, the water bottle was taken out of the metabolic cage. Under anesthesia, retro‐orbital bleeding was used to obtain blood samples. An OsmoPRO Multi‐Sample Micro‐Osmometer (Advanced Osmometer Instruments) was used to measure the osmolality of urine and plasma. ELISA kits (Enzo) were used to measure plasma vasopressin in accordance with the manufacturer's instructions. A flame photometer was used to measure the concentration of Na^+^ in plasma. Mice weighing around 30 g were given an intraperitoneal injection of either 0.5 mL of 2 M mannitol or the vehicle (water) for the mannitol injection.

### 2.6 RNA Isolation and RT‐qPCR

The RNAqueous‐Micro Total RNA Isolation Kit was used to extract RNA from OVLT neurons, and Trizol (Invitrogen) was used to extract RNA from OVLT tissue. After that, RNA samples were processed with TURBO DNase (catalog AM1907, Invitrogen) before cDNA synthesis was carried out using a Bio‐Rad iScript cDNA Kit. After diluting cDNA, real‐time qPCR was carried out using the Bio‐Rad SYBR mix. The following primers were used: SPAK F, CCCATCTGCAGGGACGC, and R, TACGTTGGGATGGCTGCATT; OSR F, AGAAGGGAAAGTTTGGCCCG, and R, GGCTGCGACCGACCTC.

### 2.7 OVLT Neuron Isolation and Electrophysiology

As previously mentioned, OVLT neurons were isolated [21]. Briefly, mice were anesthetized and killed by decapitation, and their brains were promptly taken out and put in a cold (4°C) oxygenated HBSS solution (14 175 095, Gibco; pH 7.3). After being dissected, the OVLT‐containing tissues (~1 mm^3^) that were situated dorsal and rostral to the third ventricle's preoptic recess were incubated for 30 min at 37°C in HBSS solution containing 2 mg/mL papain (LS003119, Worthington). They were then rinsed in HBSS solution without enzymes and filtered through a 100 μm filter mesh. The resultant pellet was suspended in 10% FBS + DMEM after the solution was centrifuged at 100 ***g*** for 5 min. After 2 h of plating the suspension onto coverslips coated with laminin and PDL, cells were utilized for patch clamping.

### 2.8 Whole‐Cell Recordings Were Performed as Described Previously [22]

Axopatch 200B patch‐clamp amplifier and Pulse software (Molecular Devices) were used to amplify and record currents and potentials. Low‐pass currents were filtered at 2 kHz and sampled every 0.1 milliseconds. Data acquisition was performed using a pClamp9.2 program (Axon Instrument Inc.) and analysis using Prism (V8.0) software (GraphPad Software, San Diego, CA, USA). For recording neurons, the pipette resistance was approximately 5–7 MΩ when filled with the pipette solution. Whole‐cell access resistance was less than 20 MΩ. For HEK293 cells, the pipette resistance was ~2–3 MΩ when filled with the pipette solution. Whole‐cell access resistance was < 10 MΩ. Voltage clamp experiments to record K^+^ currents were performed in whole‐cell patch mode with extracellular solution containing 140 mM NaCl, 1.8 mM CaCl_2_, 5 mM KCl, 1 mM MgCl_2_, 5.5 mM glucose, 0.33 mM NaH_2_PO4, 10 mM HEPES (pH 7.4 with NaOH); intracellular solution contained 130 mM K‐acetate (Kac), 1 mM MgCl_2_, 2 mM ATP‐Mg, 10 mM EGTA, 0.1 mM GTP, 10 mM HEPES (pH 7.2 with KOH), 4 mM CaCl_2_ (100 nM free CaCl_2_ buffering determined with Maxchelator, Stanford University). The voltage step protocol includes holding at −60 mV, and 200 (neurons) or 400 (HEK293) ms steps from −80 mV to +80 mV in +20 mV increments. To test the effect of hypertonicity on the K^+^ currents in OVLT neurons or HEK293 cells, an additional 5 mM NaCl was added to increase the osmolality in the bath solution by approximately 10 mOsm.

### 2.9 Stereotaxic Injection

Either a 4% isoflurane inhalation or a ketamine/xylazine injection was used to anesthetize the mice prior to stereotaxic injections. The mice were put in the stereotaxic frame (IVM‐3000, Scientifica) after their scalps were shaved. Throughout the procedure, each mouse's face was submerged in an anesthetizing mask that contained 1.5% isoflurane for isoflurane anesthesia. By placing a physiological‐biological temperature controlling pad (TMP5b, SuperTech Instruments) beneath each mouse's body, the temperature was kept between 34°C and 36°C. Two ear bars were used to immobilize the head, 75% ethanol was used to sterilize and disinfect the surgical scalp, and an optical gel was used to shield the animal's eyes. They positioned and inserted a 10 μL NanoFil syringe (World Precision Instruments) with a 35‐gauge beveled metal needle. A nanopump controller (KD Scientific) was used to administer the infusions for 2 min at a rate of 0.1 μL/min. Following the injection, the needle was held for 5 min before being gradually removed. Every animal was given at least 7 days to fully recuperate.

The coordinates for injections were as follows: bilateral PVN (AP, −0.8 mm; ML, ±0.25 mm; DV, 5.3 mm; relative to the bregma, modified from Nomura et al. [23]); OVLT (AP, 0.76 mm; ML, ±0 mm; DV, 4.15 mm, relative to bregma [24]). The retrograde AAV‐Cre and GFP‐AAV‐Cre viruses were injected at 0.2 μL, approximately 1 × 10^13^ virus genome/mL.

### 2.10 Ex Vivo Patch‐Clamp Recording in OVLT Slices

Acute brain slices were prepared at least 3 weeks after the virus injection, and recording was performed as previously described [25]. All animals were anesthetized with isoflurane and then sacrificed by beheading. Mouse brains were quickly removed, and using a microslicer (DTK‐1000, Dosaka), coronal brain sections with the OVLT that were 300 μm thick were cut in an ice‐cold, oxygenated (95% O_2_ and 5% CO_2_) sucrose‐based solution that contained the following ingredients: 87 mM NaCl, 75 mM sucrose, 25 mM NaHCO_3_, 10 mM glucose, 7 mM MgCl_2_, 2.5 mM KCl, 1.25 mM NaH_2_PO_4_, and 0.5 mM CaCl_2_. After being sectioned, brain slices from virus‐injected mice were immediately incubated for 30 min at 34°C in the holding chamber with the same solution. They were then kept in the same chamber at room temperature (23°C ± 2°C) until they were needed. During experiments, slices were placed in a recording chamber and superfused with oxygenated artificial cerebrospinal fluid containing 125 mM NaCl, 25 mM NaHCO_3_, 25 mM glucose, 2.5 mM KCl, 2 mM CaCl_2_, 1.25 mM NaH_2_PO_4_, 1 mM MgCl_2_, and the following synaptic blockers: 2 mM kynurenic acid (catalog K3375, Merck KGaA), 1 μM SR95531 (catalog ab120042, Abcam), and 1 μM CGP55845 (catalog 1248, Tocris Bioscience) at 23°C ± 2°C using a temperature controller (TMP5b, Supertech Instruments). PVN‐projecting OVLT neurons were confirmed by red fluorescence and visually selected under an infrared and differential interference contrast (IR‐DIC) microscope (BX51WI, Olympus) equipped with an infrared‐sensitive charge‐coupled device camera (C7500‐50, Hamamatsu). Whole‐cell recordings were performed with a digitizer‐equipped (Digidata 1440A, Molecular Devices) amplifier (Axopatch 200B or 700B, Molecular Devices). Recording electrodes with a pipette resistance of 4–6 MΩ were prepared from borosilicate glass with a filament (OD, 1.5 mm; ID, 0.86 mm; GC150F‐7.5, Harvard Apparatus) using a vertical puller (PC‐10, Narishige) and a microforge (MF‐830, Narishige). Recording electrodes were filled with the low Cl^−^ internal solution containing the following: 136.8 mM K‐gluconate, 10 mM HEPES, 7.2 mM KCl, 7 mM Na_2_‐phosphocreatine, 4 mM MgATP, 0.5 mM Na_3_GTP, 0.2 mM EGTA, and 0.4% (wt/v) biocytin (pH 7.3 adjusted with KOH). For all the recordings, the pipette capacitance was fully compensated, and series resistance was compensated to approximately 80% (bandwidth, 1–2 kHz) in the current‐clamp configuration. Signals were low‐pass filtered at 2 kHz using a 4‐pole Bessel filter and sampled at 10 kHz.

### 2.11 Statistics

Data are presented as mean ± SEM. Experimental n number is illustrated by scatter plot. Statistical comparisons between two groups of data were made using 2‐tailed unpaired Student's *t* test or paired t test as specified. Multiple comparisons were determined using 2‐way ANOVA followed by Šidák's or Tukey's multiple‐comparison tests. Electrophysiological data were analyzed using Clampfit 10.7 (Molecular Devices). The number of action potential spikes (no. AP) was analyzed using 10‐s bins, and z scores were calculated by normalizing to the SD of no. AP during 5‐min baseline before elevating extracellular tonicity [*z* = (no. AP—mean no. AP_baseline_)/SD no. AP_baseline_]. The cells with an average z score after hypertonicity stimulation (Δz score) larger than 0.5 were classified as the stimulation‐responsive (R) cells. Statistical significance between the responsiveness of each group was tested using two‐tailed Fisher's exact test. *p* values of less than 0.05 were considered significant.

### 2.12 Study Approval

All experimental procedures conform to the Guide for the Care and Use of Laboratory Animals (National Academies Press, 2011) and were approved by the Institutional Animal Care and Use Committees at the University of Iowa Carver College of Medicine and National Yang Ming Chiao Tung University.

## 3 Results

### 3.1 WNK1 Mediates Hyperosmolality Stimulation of Kv3.1 Channels in OVLT Neurons

**FIGURE 1:**
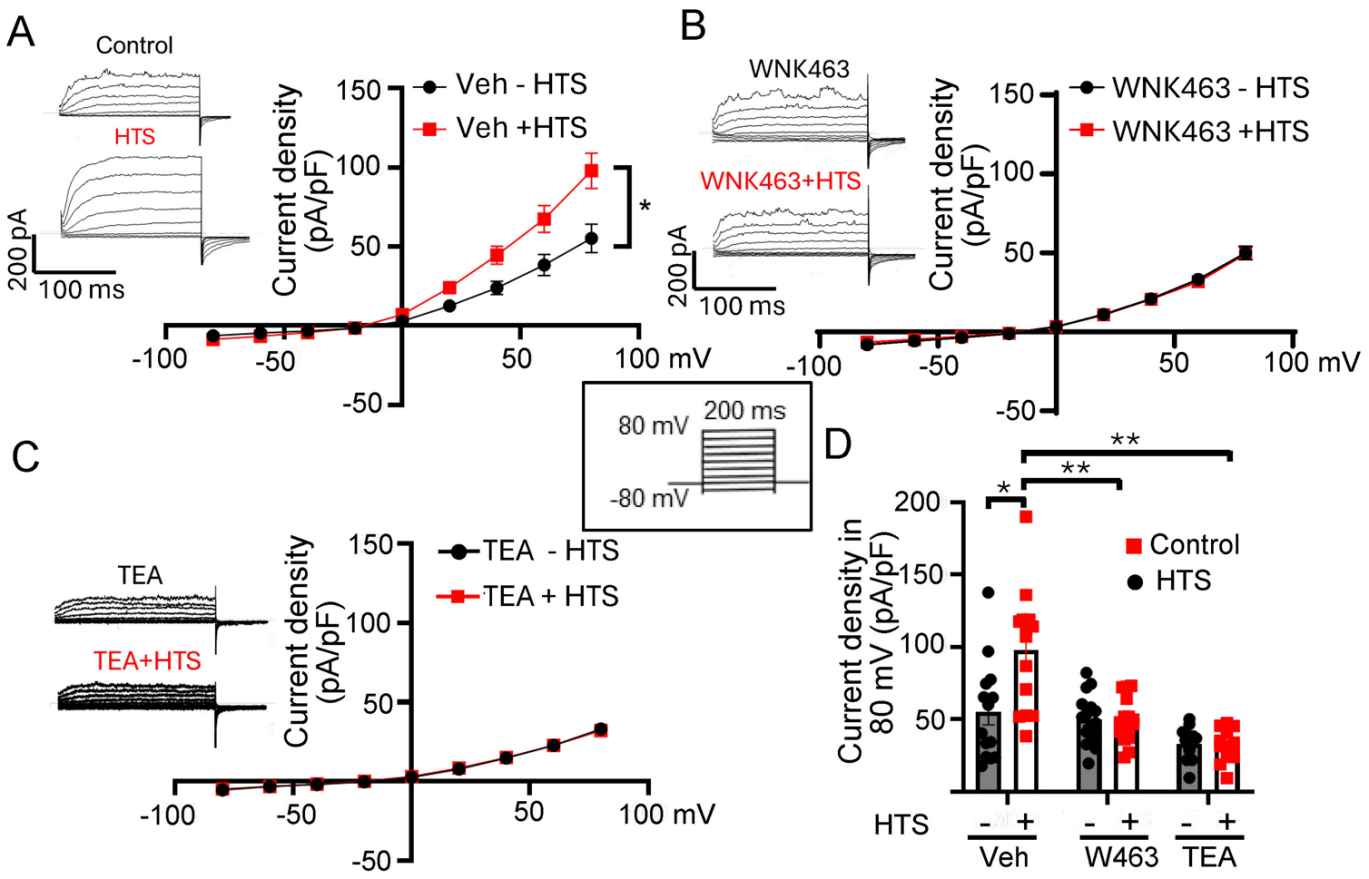
Hypertonicity stimulation of Kv3.1 channels through WNK1 in OVLT Neurons. (A–D) Ruptured whole‐cell K^+^ current in freshly isolated OVLT neurons was recorded by voltage‐clamp. The holding potential was −60 mV, and currents elicited by the membrane potential steps in +20 mV for 200 ms ranged from −80 mV to +80 mV. (A) Representative step currents and average current–voltage (I–V) relationship curve in cells pretreated with vehicle (Veh) and with or without hypertonic stimulation (HTS; Δ5 mM NaCl). The Y‐axis is current density normalized to cell membrane capacitance (pA/pF). The X‐axis is membrane voltage, ranging from −100 to +100 mV. (B) OVLT neurons were preincubated with pan‐WNK kinase inhibitor (WNK463, 10 μM) for 3 h before and after hypertonicity. (C) OVLT neurons were preincubated with TEA (3 mM) before and after hypertonicity. (D) Bar graph (pA/pF at +80 mV, mean ± SEM) showing HTS increases K^+^ current (*p < 0.05, by paired two‐tailed *t* test), but treatment with WNK463 and TEA significantly decreases the K^+^ current (**p < 0.05, by one‐way ANOVA). The scatter plots indicate the number of cells, 15–22 cells.

To investigate the downstream mechanisms for WNK1 mediating central osmosensing we first employed an in vitro model using freshly isolated OVLT neurons to study responses to hypertonicity. Whole‐cell K^+^ currents were recorded in isolated neurons before and after the application of an additional 5 mM NaCl to the bath (i.e., Δ5 mM NaCl; “HTS” for hypertonic stimulation) (Figure 1). The effective osmolality is also known as tonicity. The hypertonicity‐induced responses were examined in three different settings: in the presence of a chemical pan‐WNK inhibitor WNK463, Kv3.1 inhibitor tetraethylammonium (TEA) or vehicle control. The extracellular and pipette [K^+^] were 5 and 130 mM, respectively, with the calculated equilibrium potential for K^+^ (E_K_) at −80 mV. Currents were measured under voltage‐clamp with the membrane holding potential at −60 mV and a 200 ms step depolarization in +20 mV increments from −80 to +80 mV. At the baseline without HTS, the current–voltage (I‐V) relationship of currents revealed voltage‐dependent activation of currents (outward K^+^ currents) at −15 mV. The I‐V relationship is characteristic of Kv3.1 channels. HTS increased the K^+^ current in control freshly isolated OVLT neurons treated with vehicle (Figure 1A,D). Pretreatment with WNK463 completely blunted HTS stimulation of K^+^ currents (Figure 1B,D), supporting the notion that WNK1 mediates the increases in K^+^ currents in response to the hypertonicity stimulation. Pretreatment with TEA was employed to further differentiate Kv3.1 from other K^+^ channels. Whereas TEA applied intracellularly effectively blocks K^+^ permeation through all K^+^ channels, extracellular application of TEA selectively blocks Kv3.1 channels with an IC50 of ~300 μM [26]. We found that TEA reduced the baseline K^+^ current. The hypertonicity‐induced increases in K^+^ current in OVLT neurons were completely inhibited by 3 mM TEA (Figure 1C,D). The findings that currents are inhibited by extracellular TEA and the activation threshold at −15 mV support that they are mediated by Kv3 channels. These results establish that hypertonicity activates WNK1 to stimulate Kv3.1 in the OVLT.

### 3.2 Hyperosmolality Stimulation of Kv3.1 Channels via WNK1 Requires OSR1/SPAK

**FIGURE 2:**
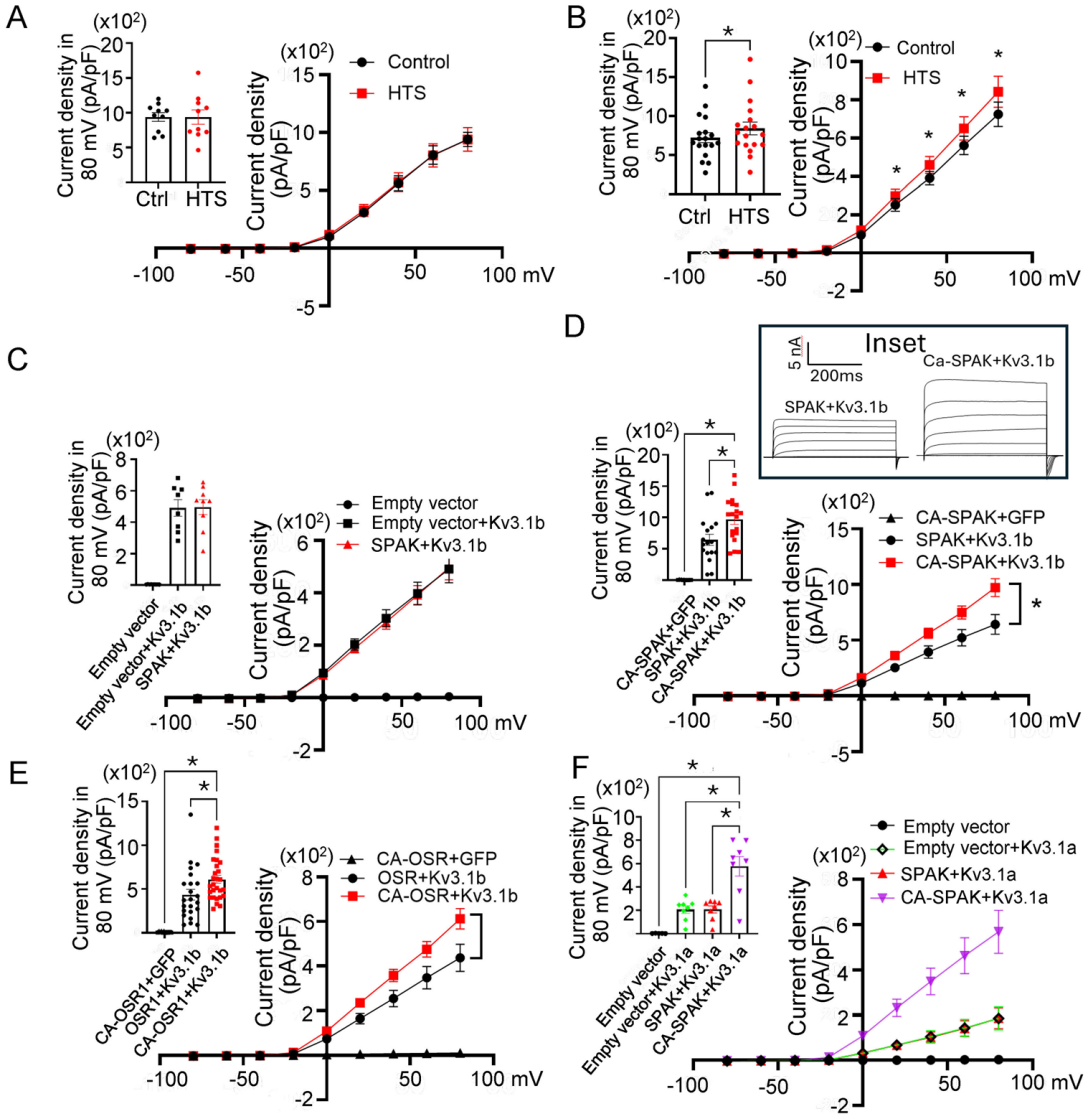
Hypertonicity stimulation of Kv3.1 channels in the reconstitution system via WNK1 requires OSR1/SPAK. (A) Whole‐cell K^+^ currents were recorded using voltage clamp in HEK293 cells transfected with WT SPAK, Kv3.1b, and empty vector (i.e., no WNK1) and subjected to without (Ctrl) or HTS. The bar graph shows the current density (pA/pF at +80 mV, mean ± SEM). (B) Whole‐cell K^+^ currents in HEK293 cells transfected with WT SPAK, Kv3.1b, and WNK1. *p* < 0.05, by paired two‐tailed *t* test. (C) Whole‐cell K^+^ currents in HEK293 cells transfected with empty vector only or with Kv3.1b, along with or without WT SPAK. Cells transfected with the vector alone showed no outward currents. (D) Whole‐cell K^+^ current in HEK293 cells transfected with GFP‐Kv3.1b or GFP alone, and with constitutively activated SPAK (CA‐SPAK) or WT‐SPAK. Inset shows current trace at different voltage steps. (E) Whole‐cell K^+^ current in HEK293 cells transfected with GFP‐Kv3.1b or GFP alone, and with constitutively activated OSR1 (CA‐OSR1) or WT‐OSR1. (F) Whole‐cell K^+^ current in HEK293 cells transfected with GFP‐Kv3.1a or empty vector, and with constitutively activated SPAK (CA‐SPAK) or WT‐SPAK. The voltage clamp protocol is the same as Figure 1, except the stimulation time is 400 ms. Bar graph (pA/pF at +80 mV, mean ± SEM). Statistical analysis in Panel A and B was performed with a paired two‐tailed *t* test; the others were performed with one‐way ANOVA, *p* < 0.05. The scatter plots indicate the number of cells, 8–30 cells.

WNK kinases may activate downstream effectors by directly phosphorylating the target or indirectly through activating other kinases including OSR1/SPAK, PI4K, and MAPK [14, 15, 16]. OSR and SPAK are members of the Ste20 kinase subfamily, share a homologous kinase domain and have similar functions. Once activated by WNK1, OSR1 and SPAK phosphorylate the target proteins. We performed reconstitution experiments in HEK cells to test the hypothesis that WNK1 activates Kv3.1 through OSR1/SPAK. We have previously found both Kv3.1 alternatively spliced isoforms, Kv3.1a and Kv3.1b, are present in OVLT [9]. We transfected cultured HEK cells with cDNA coding for Kv3.1b with or without WNK1, and/or wildtype or constitutive‐active OSR1/SPAK and recorded whole‐cell K^+^ currents before and after HTS stimulation. In cells transfected with Kv3.1b and WNK1 without co‐transfection of SPAK, HTS had no effect (Figure 2A). In contrast, cells transfected with WNK1, Kv3.1b and SPAK responded to HTS (Figure 2B). To support that hypertonicity stimulation of WNK1 is essential, we examined the effect of expressing SPAK on Kv3.1b without WNK1 or HTS and found no activation of Kv3.1b in this setting (Figure 2C). For comparison, constitutive‐active SPAK could activate Kv3.1b without WNK1 or HTS (Figure 2D). Similarly, constitutive‐active OSR could activate Kv3.1b without WNK1 or HTS (Figure 2E). The effect of activated SPAK on Kv3.1 also occurred on Kv3.1a (Figure 2F). Thus, hyperosmolality activates the WNK1‐SPAK cascade to stimulate Kv3.1.

### 3.3 Activation of OSR1/SPAK in CVOs by Osmotic Stress

**FIGURE 3:**
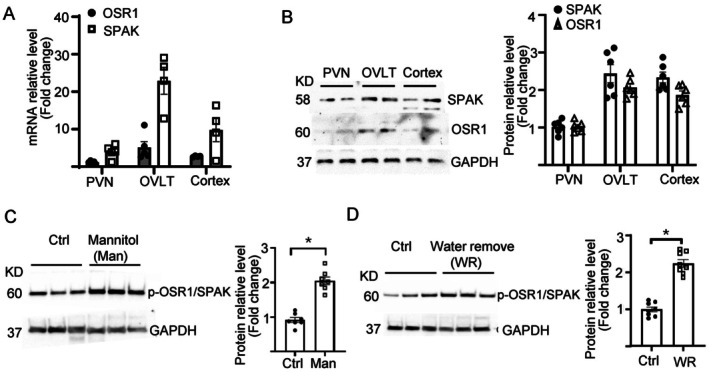
Hypertonicity increases phosphorylation of OSR1/SPAK in CVOs. (A) OSR1/SPAK mRNA levels in WT mouse brain regions were determined by qRT‐PCR. (B) Representative Western blot of SPAK/OSR1 protein in WT mouse brain regions and average abundance from multiple experiments. Total 4 experiments, one mouse in each. (C) Representative western blot and average (mean ± SEM) abundance of phosphor‐OSR1/SPAK in the OVLT isolated from WT mice 2 h after intraperitoneal injection of mannitol or vehicle (Ctrl). (D) Representative western blot and average (mean ± SEM) abundance of phosphor‐OSR1/SPAK in the OVLT isolated from WT mice with ad lib water intake or 36 h of water restriction. Statistical analysis was performed with an unpaired two‐tailed *t* test, *p* < 0.05.

We examined the expression of OSR1 and SPAK in brain regions. The transcript of OSR1 and SPAK was both present in the PVN, OVLT, and brain cortex and the OVLT had the relatively highest abundance (Figure 3A). In all 3 regions, SPAK was higher than OSR1. Protein abundance by western blot analysis confirmed higher OSR1/SPAK in the OVLT than in the cortex and PVN, and that SPAK was slightly higher than OSR1 in the OVLT (Figure 3B). WNK1 activates OSR1 and SPAK by phosphorylating serine‐325 of OSR1 and serine‐373 of SPAK. To examine the role of phosphorylation and activation of OSR1 and SPAK downstream of WNK1 for osmosensing, we measured the abundance of phosphorylated OSR1 and SPAK (p‐OSR1/SPAK) using an antibody that recognizes the specific phosphorylated serine residue. Intraperitoneal injection of mannitol or water restriction for 36 h was performed as described previously [9]. Both maneuvers increased the abundance of p‐OSR1/SPAK in isolated OVLT tissues relative to the control (Figure 3C,D). The phospho‐Ab does not distinguish between p‐OSR1 and p‐SPAK due to conservation of amino acid sequence in the epitope used for antibody production and similar molecular size of the proteins.

### 3.4 Deletion of OSR1/SPAK in CVOs Impairs Hyperosmolality Stimulation of AVP Release Causing Partial Central Diabetes Insipidus

**FIGURE 4:**
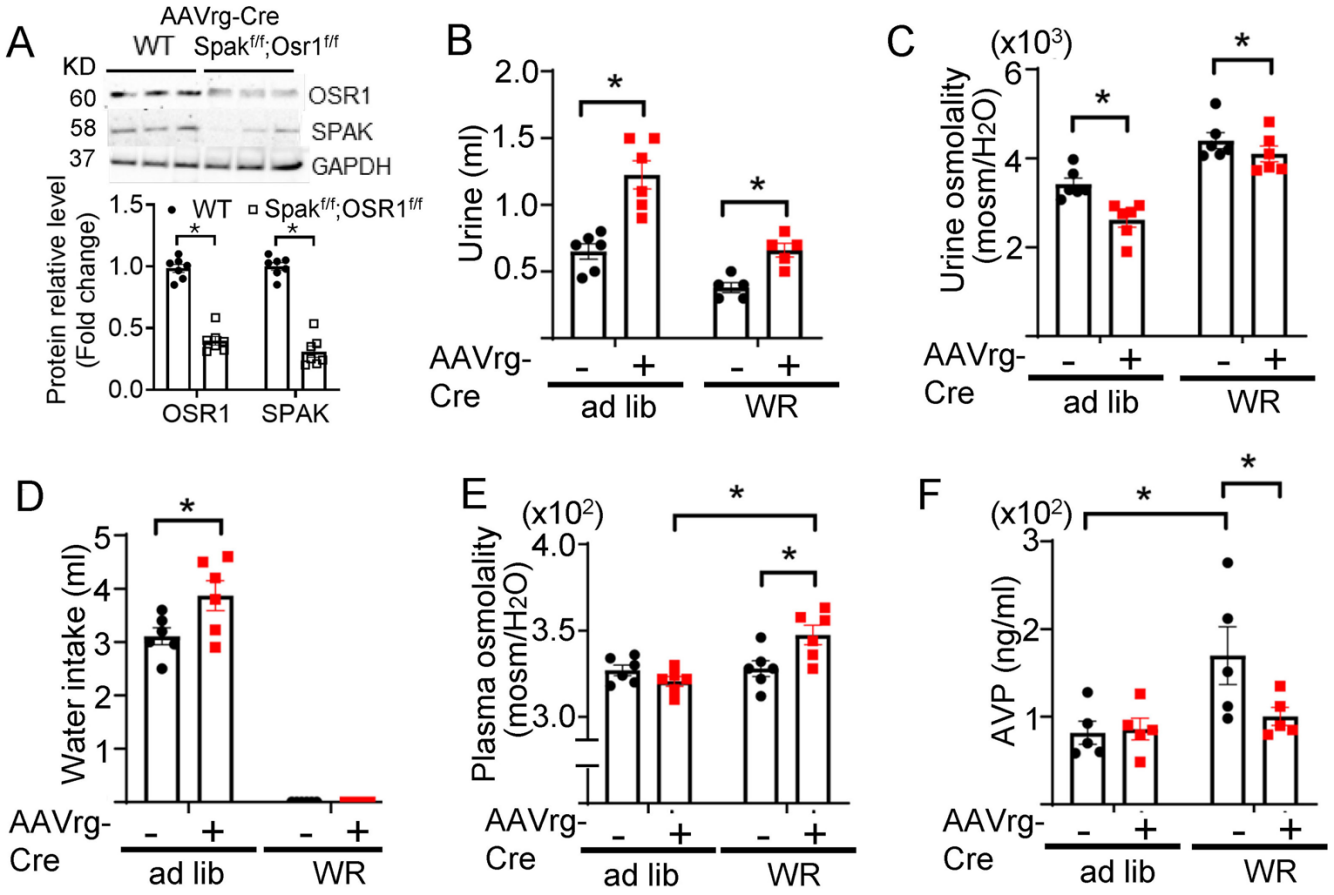
Mice deficient in OSR1/SPAK in CVOs exhibit impaired hypertonicity stimulation of AVP release and partial central diabetes insipidus. Double *Osr1* and *Spak*‐floxed mice (*Osr1* ^*f/f*^; *Spak* ^*f/f*^) received an injection of retrograde AAV virus containing Cre recombinase (“AAVrg‐Cre”) into the PVN. (A) Representative western blotting and average (mean ± SEM) abundance of OSR1 and SPAK in the OVLT of each experimental group. Three mice in each group were compared side by side. Each lane is one mouse. Experiments were repeated with total 6 samples shown in the bar graph. *p* < 0.05, by unpaired two‐tailed *t*‐test. (B–F) Metabolic cage studies of mice before and 13 days after virus injection. (B) urine volume, (C) urine osmolality, (D) water intake, (E) plasma osmolality, and (F) plasma AVP concentrations of *Osr1* ^*f/f*^; *Spak* ^*f/f*^ mice before (AAVrg‐Cre “–”) and after virus injection (AAVrg‐Cre “+”) during *at libitum* (ad lib) and after water restriction (WR). Statistical analyses were performed by two‐way repeated ANOVA with Šidák post hoc analysis. *p* < 0.05. *n* = 5–6 mice as indicated by scatter plots.

To examine the role of OSR1 and SPAK in central osmosensing for AVP release and water homeostasis, we performed metabolic cage clearance studies in mice with site‐specific deletion of the genes. All neurons in CVOs are not involved in osmolality sensing and for regulation of AVP release [9]. Osmosensory neurons in CVOs project to the AVP‐producing magnocellular neurosecretory neurons in the PVN and SON. For selectively deleting OSR1 and SPAK in PVN‐projecting osmosensory neurons in CVOs, we employed the retrograde neuronal tracing approach. Mice homozygous for doubly *Spak*‐ and *Osr1*‐floxed (*Spak* ^f/f^; *Osr1* ^f/f^) received injection of the retrograde AAV virus carrying Cre recombinase (AAVrg‐Cre) into the PVN. WT mice in which the PVN was injected with the same retrograde AAVrg‐Cre were used as controls. The experimental protocol and timeline are illustrated in Figure S1. Western blotting and immunofluorescent staining confirmed that SPAK and OSR1 were markedly reduced in OVLT neurons in the double‐floxed mice (but not in WT) in which the PVN was injected with the retrograde AAVrg‐Cre (Figure 4A; Figures S2A,B and S3). Under ad lib water access, 24‐h urine volume was significantly increased in double‐floxed mice that received AAVrg‐Cre injection (Figure 4B, labeled “+”) compared to the same group of mice before injection (“–”). Along with the increases in urine output, the urine osmolality was significantly lower in mice after vs. before injection (Figure 4C). The increases in urine volume and decreases in urine osmolality persisted during water restriction (“WR”) (Figure 4B,C), indicating that the changes were not due to polydipsia. Plasma osmolality was not significantly different in double‐floxed mice with AAVrg‐Cre virus vs. before virus injection during ad lib water access presumably due to the compensatory water intake in double *Osr1*/*Spak*‐deleted mice (Figure 4D,E). Supporting the notion, plasma osmolality was increased under water restriction (Figure 4E). Water restriction increased circulating AVP levels in double‐floxed mice without AAVrg‐Cre injection and OSR1/SPAK deletion abolished the WR‐induced AVP rise (Figure 4F), reflecting impairment in hypertonicity‐stimulated AVP release.

**FIGURE 5:**
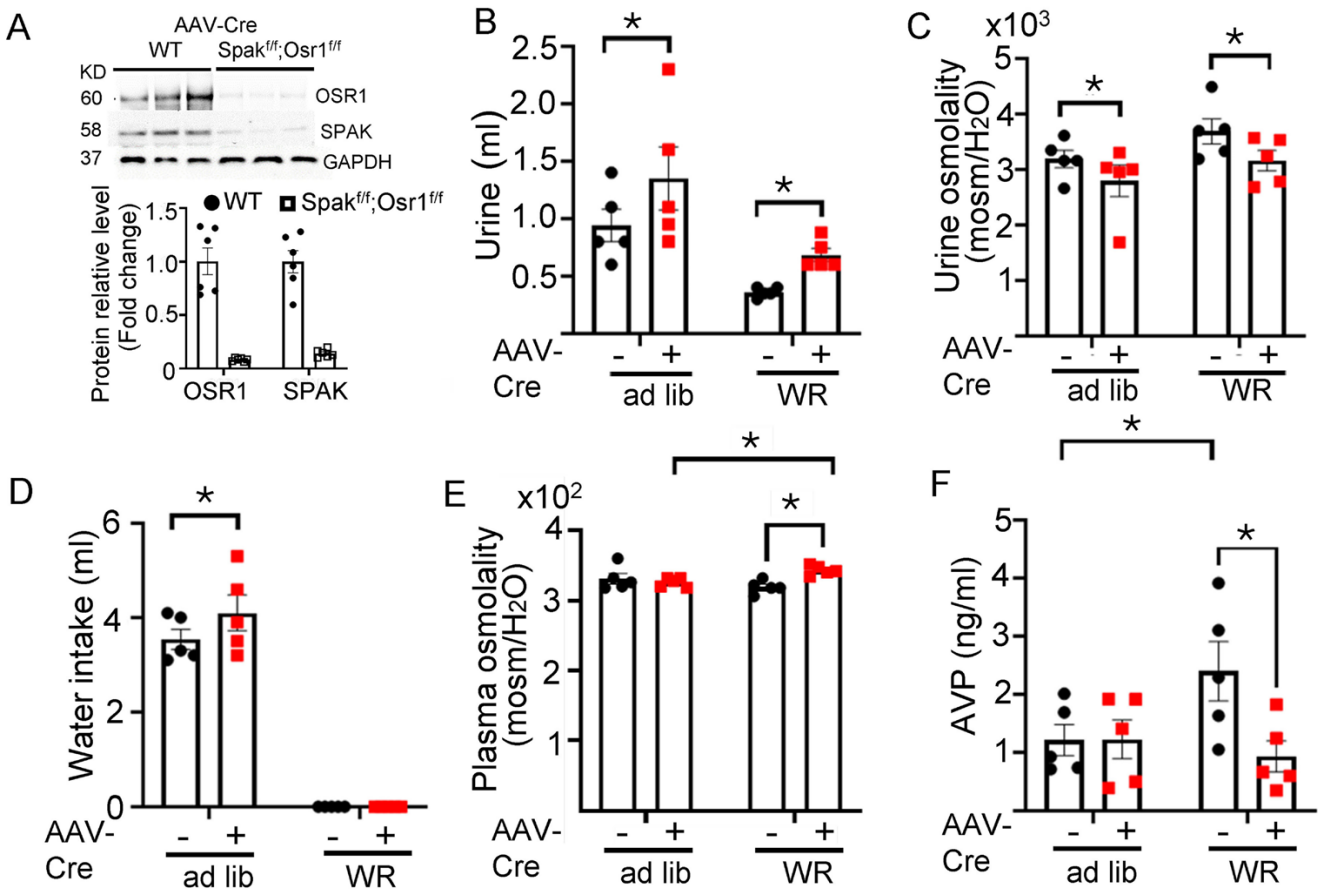
Deletion of OSR1/SPAK in OVLT impairs hypertonicity stimulation of AVP release, causing partial central diabetes insipidus. Homozygous *Osr1* ^*f/f*^; *Spak* ^*f/f*^ mice received an injection of AAV‐Cre virus or vehicle directly into the OVLT. (A) Representative western blot and average (mean ± SEM) abundance of OSR1 and SPAK protein in the OVLT of WT or *Osr1* ^*f/f*^; *Spak* ^*f/f*^ mice after AAV‐Cre virus injection. (B–F) Metabolic cage studies of these mice were conducted before and 13 days after injection. (B) urine volume, (C) urine osmolality, (D) water intake, (E) plasma osmolality, and (F) AVP levels during *ad libitum* (ad lib) and after water restriction (WR). Statistical analyses were performed by two‐way repeated ANOVA with Šidák post hoc analysis. *p* < 0.05. *n* = 5–6 mice as indicated by scatter plots.

To exclude that the injection of AAV virus induces unintended effects, we injected non‐retrograde AAV‐Cre virus into the PVN of double‐floxed mice. Double‐floxed mice received PVN injection of non‐retrograde AAV‐Cre virus were not different before and after injection with regards to water homeostasis (Figure S4A–C). To further support the above findings using the retrograde virus‐mediated gene deletion through PVN, we performed additional experiments by injecting non‐retrograde Cre‐carrying virus directly into the OVLT of double‐floxed mice. Successful injection into the OVLT and deletion of OSR1 and SPAK by this approach was validated (Figure 5A and Figure S2C,D). In contrast to experiments by injecting into the PVN, injecting the non‐retrograde Cre‐carrying virus into the OVLT directly confirmed that OSR1 and SPAK deficiency in the OVLT produced partial central diabetes insipidus phenotypes featured by relative polyuria and urine hypotonicity that persisted in water restriction, and impaired AVP release in response to water restriction (Figure 5B–F). Moreover, injecting non‐retrograde Cre‐carrying virus into the OVLT of WT mice did not produce phenotypes (Figure S4D–F). Overall, the polyuria in mice with double OSR1 and SPAK in CVOs is similar to that in mice with WNK1 deletion (~44% increases in urine volume in Figure 5B vs. ~35%–47% increases in Figures 2B and 6C in ref. [9]).

**FIGURE 6:**
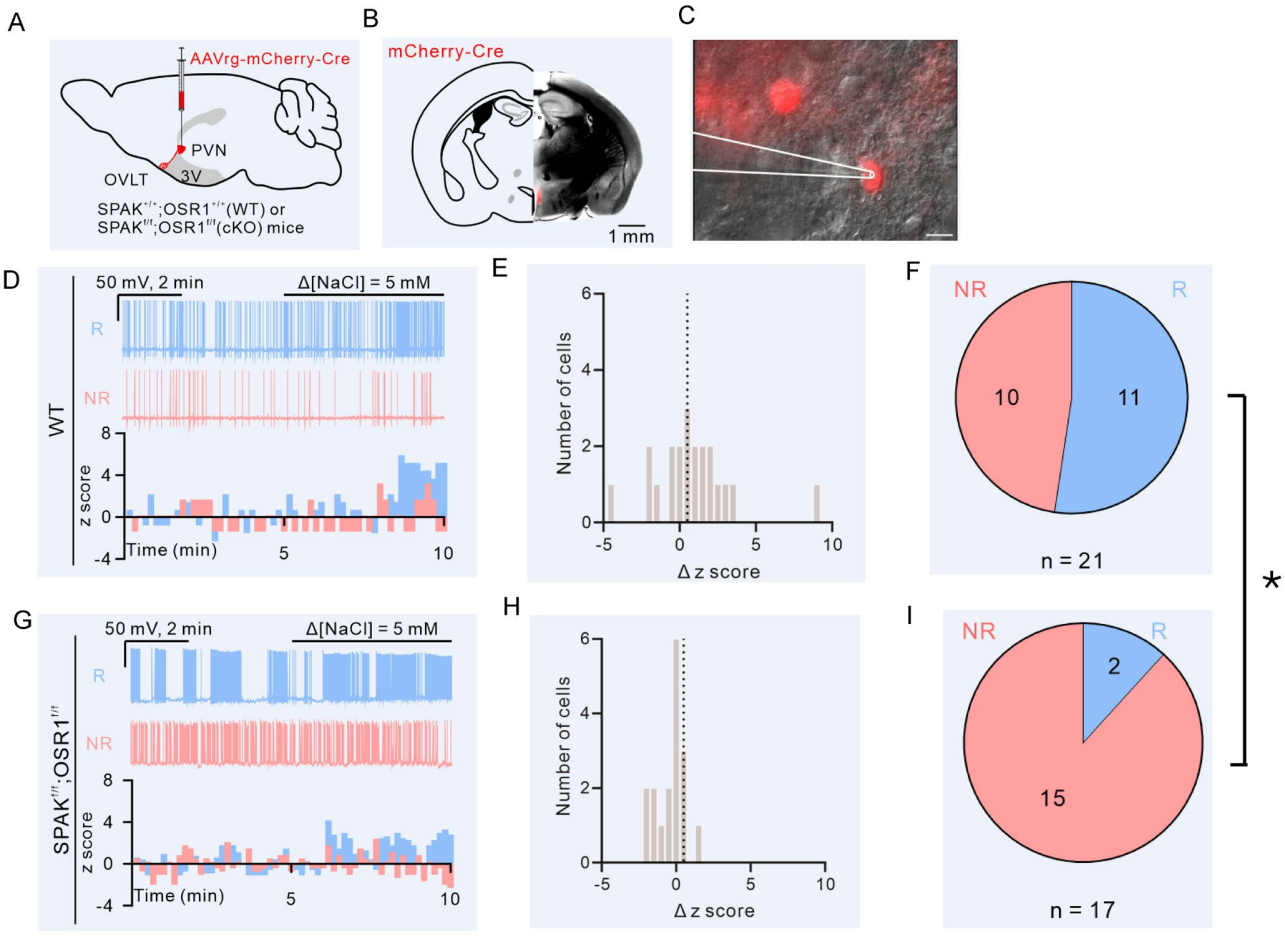
Hypertonicity‐induced action potential firing in OVLT neurons in isolated brain slice recording. (A) Schematic of the virus‐mediated deletion of OSR1 and SPAK in PVN‐projecting OVLT neurons via injection of Cre‐expressing retrograde virus into the PVN. (B) Representative coronal section of the mouse brain injected with Cre‐expressing virus at the PVN region. Scale bar: 1 mm. (C) Overlay of epifluorescence and DIC images showing Cre‐expressing neurons in the OVLT region. Scale bar: 10 μm. A recording pipette attached to a Cre‐expressing cell was illustrated. (D) Top: Representative traces of spontaneous firing recorded from a neuron responsive to 5 mM NaCl stimulation (NaCl‐R neuron “R”, cyan trace) and a NaCl‐NR (non‐responsive) neuron (“NR”; red trace) in the WT mice. Bottom: Histogram of z score from the representative NaCl‐R and NaCl‐NR cells. (E) Distribution of the Δz score in response to 5 mM NaCl stimulation of all recorded neurons in WT mice. The dashed line indicates 0.5. (F) Pie chart showing distribution of NaCl‐R and NaCl‐NR PVN‐projecting OVLT neurons in WT mice. (G) Top: Representative traces of spontaneous firing recorded from a NaCl‐R neuron and a NaCl‐NR neuron in *Osr1* ^*f/f*^; *Spak* ^*f/f*^ mice. Bottom: Histogram of z score from the representative NaCl‐R and NaCl‐NR cells. (H) Distribution of the Δz score in response to 5 mM NaCl stimulation of all recorded neurons in *Osr1* ^*f/f*^; *Spak* ^*f/f*^ mice. The dashed line indicates 0.5. (I) Pie chart showing distribution of NaCl‐R and NaCl‐NR PVN‐projecting OVLT neurons in *Osr1* ^*f/f*^; *Spak* ^*f/f*^ mice. *p < 0.05, between panel (F and I), two‐tailed Fisher's exact test. The WT group consists of recordings of 21 cells from 13 mice; the cKO group consists of 17 cells from 11 mice.

### 3.5 Deletion of SPAK/OSR1 Impairs Hypertonicity‐Induced Action Potential Firing in OVLT Neurons

Osmosensory neurons in CVOs fire APs in response to the increased extracellular tonicity and relay the signals to the AVP‐producing neurosecretory neurons in the PVN and SON via synaptic inputs. To address the involvement of SPAK and OSR1 in hypertonicity‐induced spontaneous firing in PVN‐projecting OVLT neurons, we performed ex vivo recordings on the OVLT‐containing brain slices. The PVN‐projecting OVLT neurons were identified by the retrogradely expressed mCherry signals (Figure 6A–C). Spontaneous action potentials from OVLT neurons were recorded under current‐clamp configuration with the baseline membrane potentials at approximately −50 ± 5 mV. The bath solution contained synaptic blockers kynurenic acid (KA), SR85591, and CGP55845 to isolate synaptic inputs. In OVLT neurons of Cre‐expressing WT mice, application of HTS (Δ[NaCl] = 5 mM) promoted spontaneous spike generation in approximately 50% of the recorded neurons (11 out of 21 cells; Figure 6D–F). In contrast, only approximately 11% (2 out of 17 cells; Figure 6G–I) of the double *Osr1*‐ and *Spak*‐deleted OVLT neurons exhibited increased action potential firing in response to the hypertonic stimulation (*p = 0.0151, two‐tailed Fisher's exact test between Figure 6F,I). These data support the involvement of SPAK and OSR1 proteins in osmolality sensing and hypertonicity‐promoted spontaneous spike generation in OVLT neurons ex vivo.

### 3.6 Kv3.1 Deletion in CVOs Abolishes Hypertonicity‐Stimulated AVP Release and Water Homeostasis

**FIGURE 7:**
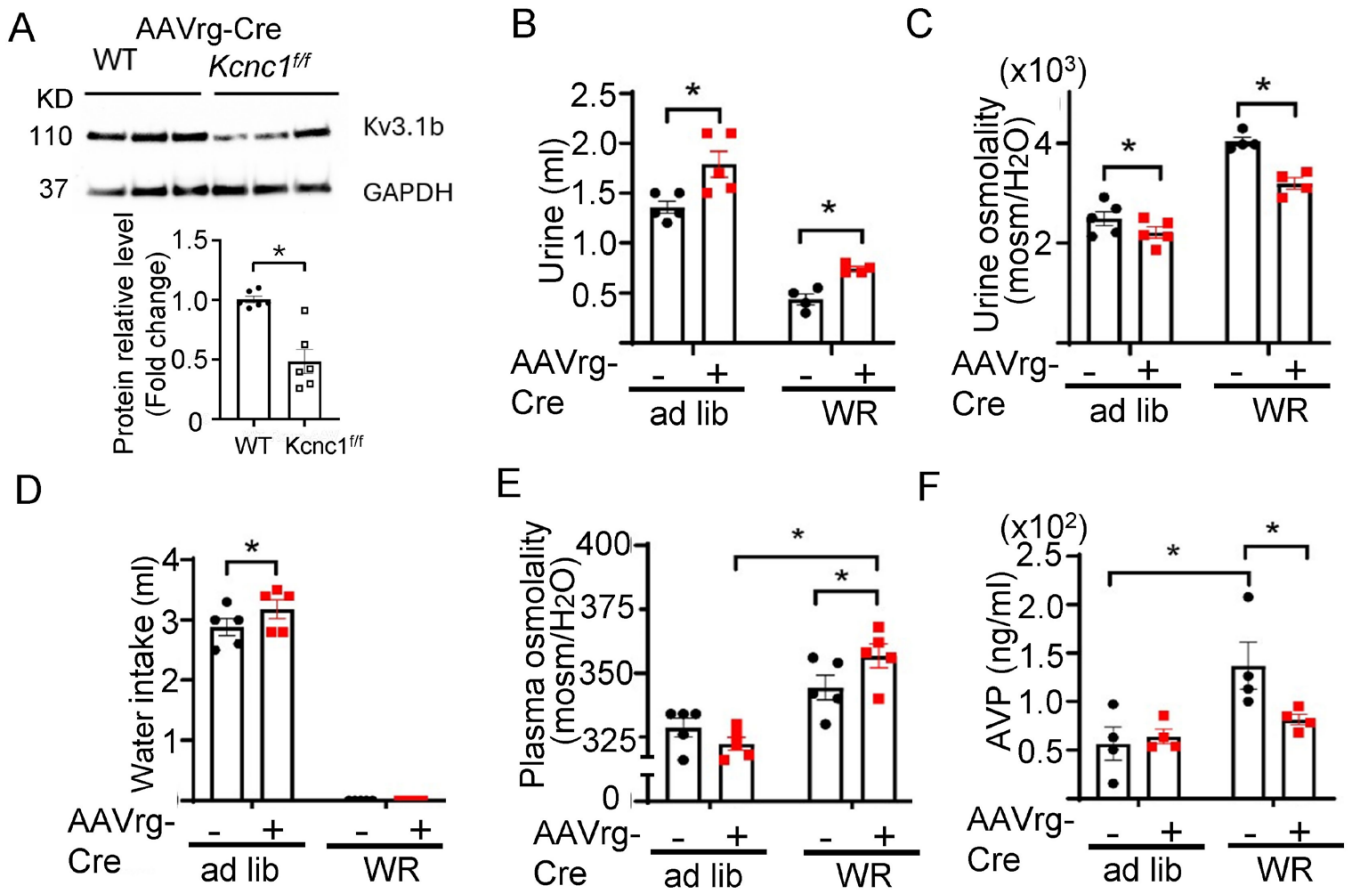
Hypertonicity‐stimulated AVP release and water homeostasis in mice lacking Kv3.1 K^+^ channels is impaired in CVOs. Mice homozygous for the *Kcnc1*‐floxed allele received an injection of retrograde Cre‐recombinase carrying AAV virus (“AAVrg‐Cre”) into the PVN. *Kcnc1* codes for Kv3.1. Two alternatively spliced forms, Kv3.1a and 3.1b, for the gene. (A) Representative western blot and average (mean ± SEM) abundance of Kv3.1b protein in the OVLT of WT or *Kcnc1* ^*f/f*^ mice after AAV‐Cre virus injection. Experiments consist of 5–6 mice in each group. Each point in scatter plots reflects one animal. 3 separate samples from each mouse were analyzed and averaged for each mouse. The relative protein fold change was compared by unpaired two‐tailed *t*‐test, *p* < 0.05. (B–F) Metabolic cage studies of these mice were conducted before and 13 days after virus injection. (B) Urine volume, (C) urine osmolality, (D) water intake, (E) plasma osmolality, and (F) plasma AVP concentrations of *Kcnc1* ^*f/f*^ mice before (AAVrg‐Cre “−”) and after virus injection (AAVrg‐Cre “+”) during *at libitum* (ad lib) and after water restriction (WR). Statistical analysis by two‐way repeated ANOVA with Šidák post hoc analysis, *p* < 0.05. *n* = 4–5 mice as indicated by scatter plots.

We have demonstrated in isolated OVLT neurons and HEK cells reconstituted with recombinant proteins that activation of SPAK/OSR1 by WNK1 increases Kv3.1 channel activity (Figures 1 and 2). Previously we have shown that both splice variants Kv3.1a and b are expressed in the OVLT, with the Kv3.1b transcript slightly more abundant [9]. An increase in Kv3.1 activity would increase afterhyperpolarization (AHP) and/or decrease AP width, which would lead to shortening of the refractory period of action potential and increase firing [17]. Indeed, we found that HTS decreased the AP half‐width in brain slice recordings (Figure S5). We carried out clearance studies to further investigate the role of Kv3.1 in the cascade of hypertonicity stimulation of AVP release and water homeostasis. We deleted *Kcnc1* coding for Kv3.1 in OVLT neurons by injecting a retrograde AAVrg‐Cre virus into the PVN of homozygous *Kcnc1*‐floxed (*Kcnc1* ^*f/f*^) mice. Western blot analysis revealed that the Kv3.1b protein was significantly reduced in the OVLT of *Kcnc1*‐floxed mice injected with AAVrg‐Cre vs. WT mice injected with the virus (Figure 7A). Compared to *Kcnc1* ^*f/f*^ mice before AAVrg‐Cre injection (“−”), *Kcnc1* ^*f/f*^ mice injected with AAVrg‐Cre (“+”) developed polyuria and relative hypotonic urine during ad lib water intake, which persisted during water restriction (Figure 7B,C). A compensatory increase in water intake was noted in Kv3.1‐deficient mice (Figure 7D). Plasma osmolality was not different in *Kcnc1* ^*f/f*^ mice with AAVrg‐Cre virus vs. before virus injection during ad lib water intake but became significantly different after water restriction (Figure 7E). Water restriction increased circulating AVP levels in *Kcnc1* ^*f/f*^ mice without AAVrg‐Cre injection and Kv3.1 deletion abolished the WR‐induced AVP rise (Figure 7F), reflecting impairment in hypertonicity‐stimulated AVP release. For control we showed that injecting retrograde AAVrg‐Cre virus directly into the PVN of WT mice had no phenotypes (Figure S6). Thus, Kv3.1 functions in the same osmosensing pathway as WNK1 and OSR1/SPAK.

### 3.7 Activation of OSR1 in CVOs Stimulates AVP Release With Reduction in Urine Output and Increases in Urine Osmolality

**FIGURE 8:**
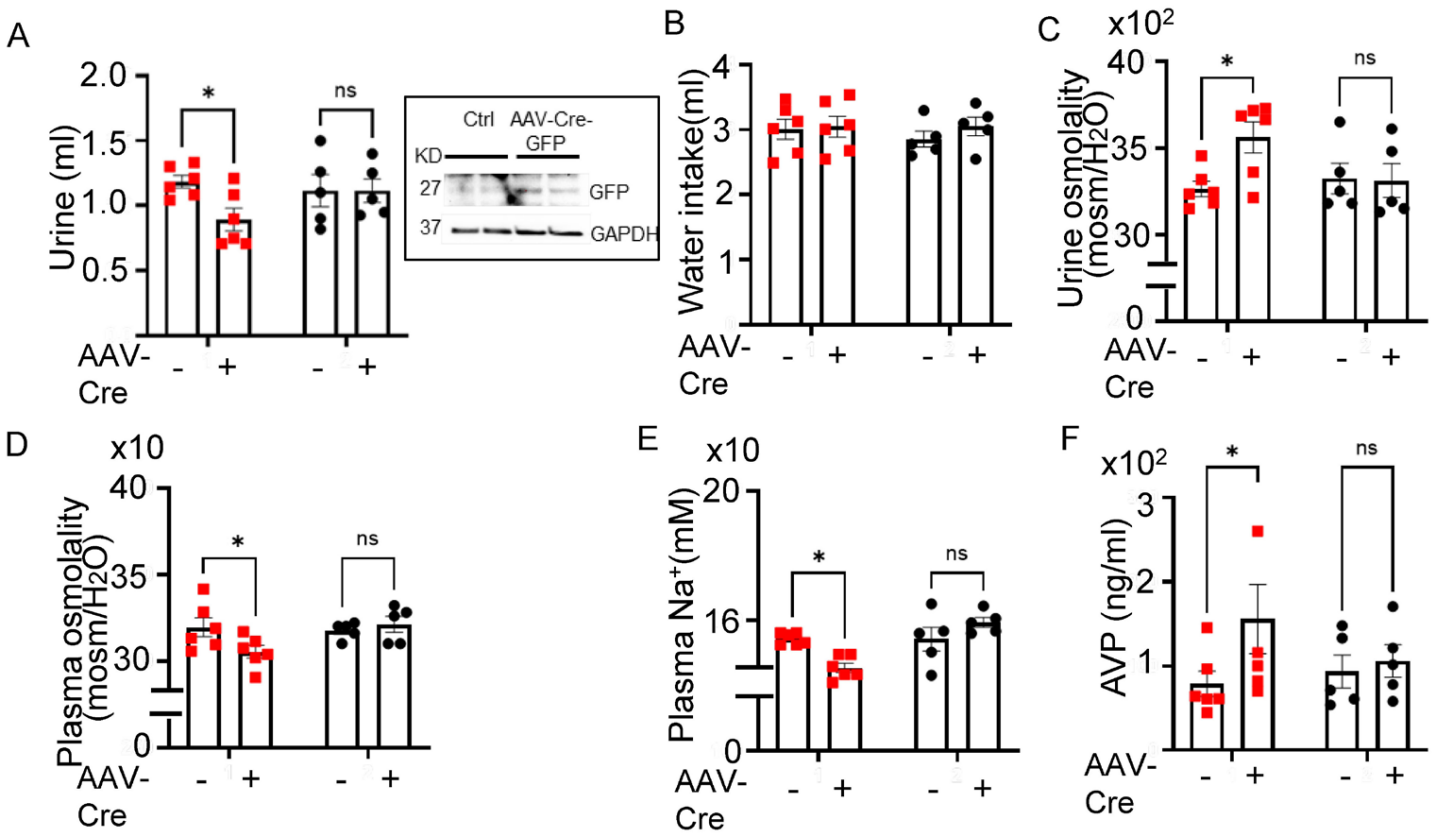
AVP release and water homeostasis in mice with gain‐of‐function of OSR1. WT mice or mice homozygous for a constitutively active *Osr1*‐knockin allele (*CA‐Osr1* ^*f/f*^) received GFP‐AAV‐Cre virus injection in OVLT. (A–F) Metabolic cage studies of these mice before (AAV‐Cre “−”) and 7 days after virus injection (AAV‐Cre “+”). (A) urine volume, (B) water intake, (C) urine osmolality, (D) plasma osmolality, (E) plasma Na^+^ concentration, and (F) plasma AVP levels of *CA‐Osr1* ^*f/f*^ mice (red squares) and WT mice (solid black). *n* = 5–6 mice, as indicated in scatter plots. Statistical analysis by two‐way repeated ANOVA with Šidák post hoc, *p* < 0.05. Inset in panel A shows GFP expression measured by western blot in the OVLT of mice receiving PBS(Ctrl) or GFP‐AAV‐Cre virus injection.

We have shown that constitutively active SPAK/OSR1 directly increased Kv3.1 currents without HTS (Figure 2) and Kv3.1 deletion inhibited AVP release (Figure 7). The results suggest that constitutive activation of SPAK/OSR1 will promote AVP release. To test the notion, we created mice with constitutive‐active OSR1 by crossing mice carrying alleles for conditionally constitutive activatable *Osr1* (*CA‐Osr1* ^*f*/f^). The constitutive activatable *CA‐Osr1* allele contains a stop codon before the mutant *Osr1* cDNA and is expressed upon Cre‐mediated excision. *CA‐Osr1* ^*f/f*^ and WT mice received injections of GFP‐AAV‐Cre virus into the OVLT. Figure S7 shows the experimental protocol and timeline. GFP immunofluorescence validated effective and widespread AAV injections into the OVLT (Figure S8A–D). Western blotting confirmed that GFP‐Cre expression in the OVLT of *CA‐Osr1* ^*f/f*^ as well as *CA‐Osr1* ^*f/f*^ mice received vehicle injection (PBS) (Figure 8A). Urine volume was significantly lower in *CA‐Osr1* ^*f/f*^ mice with AAV‐Cre virus vs. before virus injection (Figure 8A). In contrast, urine volume was not different before and after virus injection in WT mice. Water intake was not different among all study groups indicating that the decreases in urine output are independent of water intake (Figure 8B). The urine osmolality was proportionally increased along with the decreases in urine volume (Figure 8C). Plasma osmolality and Na^+^ concentration were significantly decreased and AVP levels increased in mice with constitutive‐active OSR1 (Figure 8D–F). None of the above measured parameters were different in WT mice before and after injection of AAV‐Cre. Thus, constitutive activation of OSR1 kinase activity in the OVLT increases AVP release and mice with increases in the kinase activity manifest the phenotype resembling patients with SIADH.

## 4 Discussion

The release of the antidiuretic hormone AVP is essential for the maintenance of water homeostasis for terrestrial animals. The osmosensory neurons in the brain play a central role in the process. Increases in the extracellular osmolality stimulate the osmosensory neurons leading to increased synthesis and release of AVP. The machinery responsible for sensing the hypertonicity has been elusive. We recently reported that the intracellular protein kinase WNK1 is important. Mice deleted of *Wnk1* in the CVOs exhibit impaired hypertonicity‐induced AVP release and phenotypes of central diabetes insipidus [9]. In the current report we demonstrate that OSR1/SPAK acts downstream of WNK1 to regulate AVP release in response to hyperosmolality. The CVOs of mouse brain express both OSR1 and SPAK. Deletion of both *Osr1* and *Spak* impairs hypertonicity‐induced AVP release recapitulating the phenotype of *Wnk1*‐deleted mice. Moreover, mice with gain‐of‐function of OSR1 induced by the expression of a constitutive‐active *Osr1* exhibit increased AVP release without hypertonicity stimulation with phenotypes resembling those in patients of SIADH.

Among the family of WNK kinases, WNK1 is the longest with > 2000 amino acids in length and ubiquitously expressed [5, 7]. WNK kinases regulate a wide range of targets and cell functions including many ion channels and transporters [27, 28, 29, 30]. The mechanisms for WNK kinases' regulation of ion channels and transporters may involve the catalytic and non‐catalytic mechanisms. The non‐catalytic kinase‐independent mechanisms include serving as a scaffold for targets and/or their regulators. WNK1 and WNK4 interact with intersectin, a mediator of clathrin‐coated vesicle‐mediated endocytosis, to enhance the internalization of ROMK K^+^ channels [12]. WNK1 activates serum‐glucocorticoid kinase 1 (SGK1) via protein–protein interactions to enhance the activity of the epithelial Na^+^ channel ENaC [11]. Recently, Saha et al. showed that WNK1 functions as a chloride‐dependent scaffold for SGK1 and mechanistic target of rapamycin complex 2 (mTORC2) allowing the latter to phosphorylate and activate SGK1 thus increasing ENaC activity [31]. The catalytic kinase‐dependent mechanism of regulation of ion transporters by WNK kinases is exemplified by an indirect action through intermediate kinases OSR1/SPAK kinases. WNKs phosphorylate OSR1 or SPAK to activate their kinase activity. Phosphorylated and activated OSR1 or SPAK in turn phosphorylates targets including cation‐ and Cl^−^‐dependent co‐transporters NCC, NKCC and KCC [32]. The activation of the Cl^−^‐dependent co‐transporters by OSR1 and SPAK involves an RFXV/I (arginine‐phenylalanine‐any amino acid‐valine or isoleucine) motif on the transporters recognized by the conserved C‐terminus domain of SPAK/OSR1 [33]. Kv3.1 is a high‐threshold voltage‐gated K^+^ channel present in excitable tissues. Its activation shortens the action potential and the refractory period thus enhancing action potential firing [34]. We have previously shown in ex vivo brain slice recording that WNK1 activates Kv3.1 to increase AP firing in response to hypertonicity stimulation [9]. The present study further shows that Kv3.1 is a downstream target for WNK1‐mediated regulation of AVP release and that this activation of Kv3.1 by WNK1 is through OSR1/SPAK. Importantly, deletion of Kv3.1 in CVOs in mice recapitulates the central diabetes insipidus phenotype. Interestingly, neither Kv3.1a nor Kv3.1b possess the RFXV/I motif currently known to be essential for interaction with OSR1/SPAK. How OSR1/SPAK activates Kv3.1 remains unknown. It will be interesting to test the hypothesis that WNK1 serves as a scaffold for OSR1/SPAK and Kv3.1 to allow activation of Kv3.1 as is the case for mTORC2 and SGK1.

The cell membrane tension changes as cells swell and shrink in response to variations in extracellular tonicity. Membrane resident‐mechanosensitive channels have been proposed as osmolality sensors [1, 2, 3]. Intracellular proteins may also function as an osmolality sensor as water fluxes across the cell membrane alter the intracellular water content. The structural analyses reveal that the catalytic center of WNK1 and WNK3 contains bound water and Cl^−^ molecules. Both act as allosteric inhibitors of the catalytic activity and unbinding increases the activity [8, 35]. Prior in vitro and cell‐cultured studies have shown that WNK kinase activity is activated by osmotic stress ~100 mOsmo above the baseline [36]. Our findings that WNK1 is a central osmosensor within the physiological range of osmolality fluctuation illustrate the exquisite sensitivity of the system. Our findings also indicate that OSR1 and SPAK by themselves are not osmosensitive. Other intracellular protein kinases such as apoptosis signal‐regulating kinase 1 (ASK1) and c‐Jun N‐terminal kinase (JNK) have been reported to be activated by osmotic stress [37]. The impairment of AVP release in *Wnk1*‐deleted mice is partial. Moreover, *Wnk1*‐deleted mice do not exhibit defects in the thirst mechanism and respond to polyuria by increasing water intake. Osmosensory neurons in CVOs are heterogeneous [9]. Separate sets of neurons with different osmosensing mechanisms other than WNK1 may be responsible for detecting the hyperosmotic thirst. Future studies will investigate how OSR1/SPAK activates Kv3.1 and identify additional central osmosensor(s) for regulating AVP release as well as thirst.

## Author Contributions

X.J., J.X., C.‐W.Y., and Y.‐J.L. designed and conducted experiments and analyzed the data; C.‐C.L. and C.‐L.H. supervised the project. All authors wrote the initial draft and C.‐L.H. wrote the final paper. All authors reviewed and approved the final version of the submitted manuscript.

## Conflicts of Interest

The authors declare no conflicts of interest.

## Supporting information


**Figure S1:** fsb271213‐sup‐0001‐FiguresS1‐S8.pdf.

## Data Availability

The data that support the findings of this study are available in Sections 2 and 3, and/or Figures S1–S8 of this article.
