# Supplementary material for: Central Osmolality Sensing for Arginine Vasopressin Release Is Mediated by WNK1‐OSR1/SPAK‐Kv3.1 Cascade
Source: FASEB J. 2025 Nov 17;39(22):e71213. doi: 10.1096/fj.202502072RR (PMC12621171; doi:10.1096/fj.202502072RR)
Supplement: Supplementary file 1 — Figure S1: fsb271213‐sup‐0001‐FiguresS1‐S8.pdf. [file FSB2-39-e71213-s001.pdf]

Supplementary Information

Central osmolality sensing for arginine vasopressin release is mediated by WNK1-OSR1/SPAK-Kv3.1 cascade

Xin Jin,[1] Jian Xie,[1] Chia-Wei Yeh,[2,4] Yu-Jui Li,[2] Cheng-Chang Lien,[2,3] and Chou-Long Huang[1]

[1]Department of Medicine, Division of Nephrology, University of Iowa Carver College of Medicine, Iowa City, Iowa, USA; [2]Institute of Neuroscience and [3]Brain Research Center, National Yang Ming Chiao Tung University, Taipei, Taiwan; [4]Present address: Department of Molecular, Cell and Systems Biology, University of California, Riverside, Riverside, California, USA

The supplementary information contains eight supplementary figures

Supplementary Figure Legends

Supplementary Figure S1. Timeline for experiment in Figure 4, 5, and 7 in which AAV-retro-Cre and GFP-AAV-Cre virus injection was injected. Retrograde AAVrg- Cre virus or GFP-AAV-Cre virus were injected into the PVN at day 0. Prior to that urine collection was carried out at *ad lib* for 3 days, followed by 24 hr interval, and under water restriction (WR) for 1 day. Urine output was monitored for ensuing 13 days after injection. Thereafter, urine collection was carried out at *ad lib* for 3 days, 24-interval, under water restriction (WR) for a day. A 24-hr day starts from 12 noon to 12 noon next day. Blood was collected via retro-orbital route before and after virus injection (for *ad lib* or WR).

Supplementary Figure S2. Immunofluorescent staining of SPAK and OSR1 in WT and site-specific gene deleted mice. (A-B) Immunofluorescent staining shows PVN injection of AAV-retro-Cre virus into *Osr1*$^{f/f}$*;Spak*$^{f/f}$ mice resulted in deletion of SPAK and OSR1 in the OVLT compared with control experiments with injection of AAV-retro-Cre virus into the PVN of WT mice. (C-D) Immunofluorescent staining shows direct injection of the non-retrograde GFP-AAV-Cre virus into the OVLT of *Osr1*$^{f/f}$*;Spak*$^{f/f}$ mice resulted in deletion of SPAK and OSR1 in the OVLT compared with control experiments with injection of GFP-AAV-Cre virus into the OVLT of WT mice. Orange arrows show the third ventricle (3V) under the white solid line. Green arrows show OVLT between the solid line and dotted line. Scale bar: 100 μm.

Supplementary Figure S3. Western blot of OSR1 and SPAK in OVLT from control and mice with retrograde AAV-Cre-mediated deletion of OSR1 and SPAK. Mice with double *Osr1* and *Spak*-floxed mice (*Osr1*$^{f/f}$; *Spak*$^{f/f}$) received an injection of retrograde AAV virus containing Cre recombinase ("AAVrg-Cre") into the PVN as performed in Figure 4. (A) Whole un-cut western blot as from one set of experiments shown in Figure 4A. (B) Simultaneous get electrophoresis and western blot analysis of total 6 samples in each set of experiment as described in Figure 4A.

Supplementary Figure S4. Metabolic cage studies of *Osr1*$^{f/f}$*;Spak*$^{f/f}$ mice received direct injection of Cre-recombinase non-retrograde AAV virus into the PVN (A-C) or WT mice received direct injection of Cre-recombinase non-retrograde AAV virus into the OVLT (D-F). (A,D) water intake, (B,E) urine volume, and (C,F) urine osmolality before (AAV "-") and after injection (AAV "+") during *at libitum* (*ad lib*) and after water restriction (WR).

Supplementary Figure S5. Effect of HTS on action potential profile and firing. (A) Representative spontaneous action potential in OVLT neurons in isolated brain slice recorded by current clamp (see Methods) with and without HTS. Inhibition of Kv3.1 by TEA decreases the magnitude of prolonging the refractory period. (B) AP shape at baseline and after HTS. (C) AP half-width at baseline and after HTS. (D) Afterhyperpolarization potential (AHP) at baseline and after HTS. Note that HTS significantly decreases AP half-width. HTS while tends to increase AHP, it does not reach statistical significance.

Supplementary Figure S6. Metabolic cage studies of WT mice received direct injection of retrograde AAV virus containing Cre recombinase into the PVN. (A) water intake, (B) urine volume, and (C) urine osmolality before (AAVrg-Cre "-") and after injection (AAVrg-Cre "+") during *at libitum* (*ad lib*) and after water restriction (WR).

Supplementary Figure S7. Timeline for experiment with GFP-AAV-Cre virus injection into the OVLT of *CA-Osr1*$^{f/f}$ knockin mice. GFP-AAV-Cre virus were injected into OVLT at day 0. Prior to that urine collection was carried out at *ad lib* for 3 days. Urine output was monitored for ensuing 7 days after injection. Thereafter, urine collection was carried out at *ad lib* for 3 days, 24-interval. A 24-hr day starts from 12 noon to 12 noon next day. Blood was collected via retro-orbital route before and after virus injection.

Supplementary Figure S8. GFP immunofluorescence in *Osr1*$^{f/f}$*;Spak*$^{f/f}$ (A,B) or *CA-Osr1*$^{f/f}$ knockin mice (C,D) received vehicle (PBS) or GFP-AAV-Cre virus injection into the OVLT. (A,C) as controls, PBS injections showed no green fluorescence). (B,D) GFP fluorescence in the OVLT is marked by green arrow (area between white dotted and solid line. Third ventricle (3V) is marked by orange arrow. Scale bar: 100 µm.

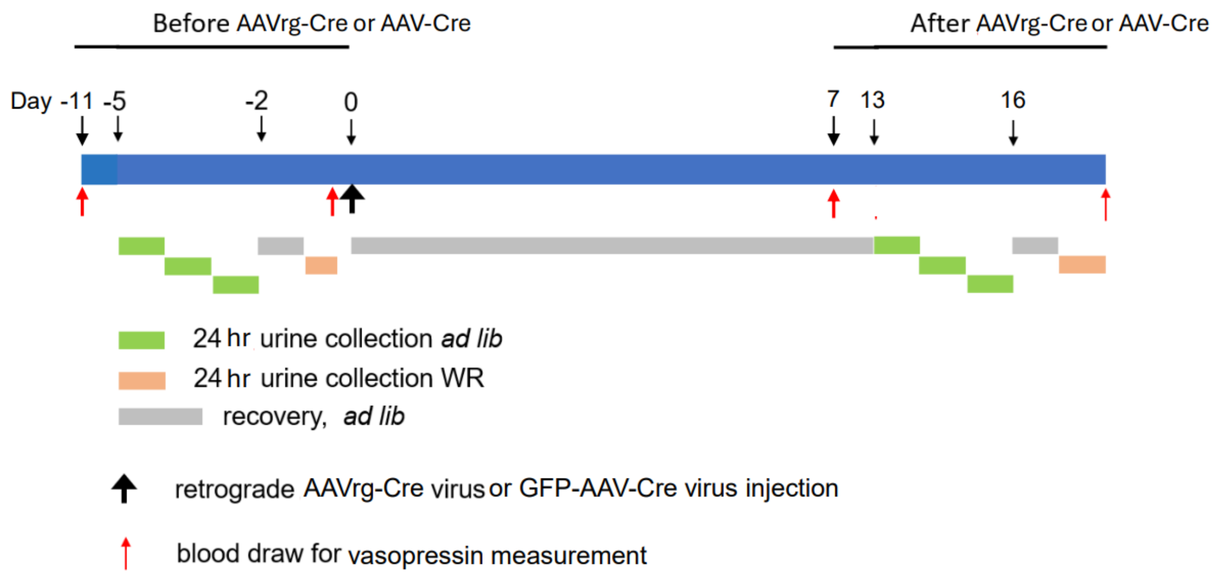

Supplementary Figure S1. Timeline for experiment in Figure 4, 5, and 7 in which AAV-retro-Cre and GFP-AAV-Cre virus injection was injected. Retrograde AAVrg- Cre virus or GFP-AAV-Cre virus were injected into the PVN at day 0. Prior to that urine collection was carried out at *ad lib* for 3 days, followed by 24 hr interval, and under water restriction (WR) for 1 day. Urine output was monitored for ensuing 13 days after injection. Thereafter, urine collection was carried out at *ad lib* for 3 days, 24-interval, under water restriction (WR) for a day. A 24-hr day starts from 12 noon to 12 noon next day. Blood was collected via retro-orbital route before and after virus injection (for *ad lib* or WR).

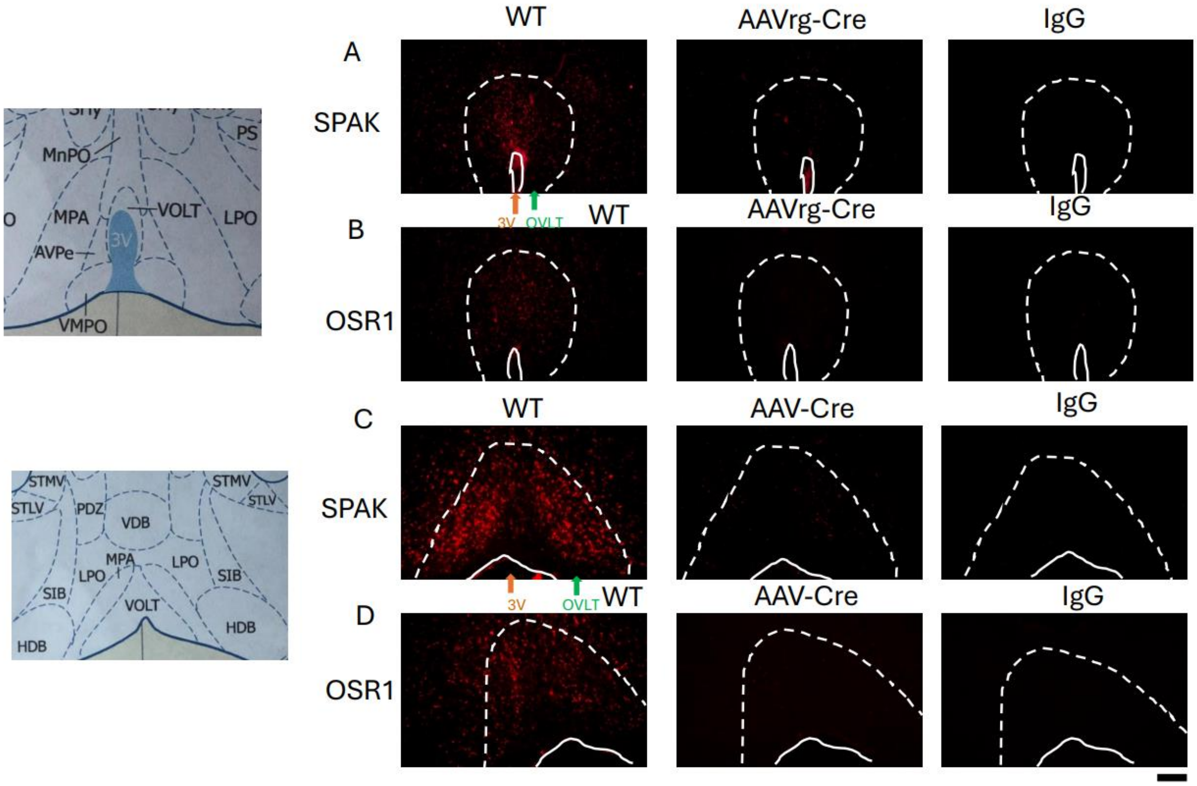

Supplementary Figure S2. Immunofluorescent staining of SPAK and OSR1 in WT and site-specific gene deleted mice. (A-B) Immunofluorescent staining shows PVN injection of AAV-retro-Cre virus into *Osr1*$^{f/f}$;*Spak*$^{f/f}$ mice resulted in deletion of SPAK and OSR1 in the OVLT compared with control experiments with injection of AAV-retro-Cre virus into the PVN of WT mice. (C-D) Immunofluorescent staining shows direct injection of the non-retrograde GFP-AAV-Cre virus into the OVLT of *Osr1*$^{f/f}$;*Spak*$^{f/f}$ mice resulted in deletion of SPAK and OSR1 in the OVLT compared with control experiments with injection of GFP-AAV-Cre virus into the OVLT of WT mice. Orange arrows show the third ventricle (3V) under the white solid line. Green arrows show OVLT between the solid line and dotted line. Scale bar: 100 μm.

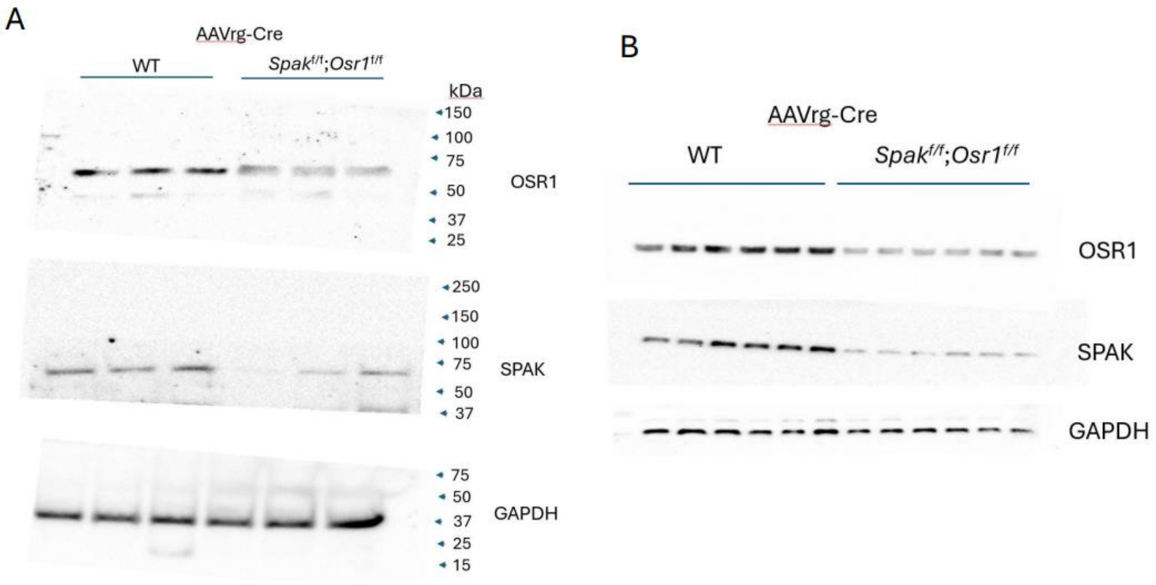

Supplementary Figure S3. Western blot of OSR1 and SPAK in OVLT from control and mice with retrograde AAV-Cre-mediated deletion of OSR1 and SPAK. Mice with double *Osr1* and *Spak*-floxed mice (*Osr1$^{f/f}$*; *Spak$^{f/f}$*) received an injection of retrograde AAV virus containing Cre recombinase ("AAVrg-Cre") into the PVN as performed in Figure 4. (A) Whole un-cut western blot as from one set of experiments shown in Figure 4A. (B) Simultaneous get electrophoresis and western blot analysis of total 6 samples in each set of experiment as described in Figure 4A.

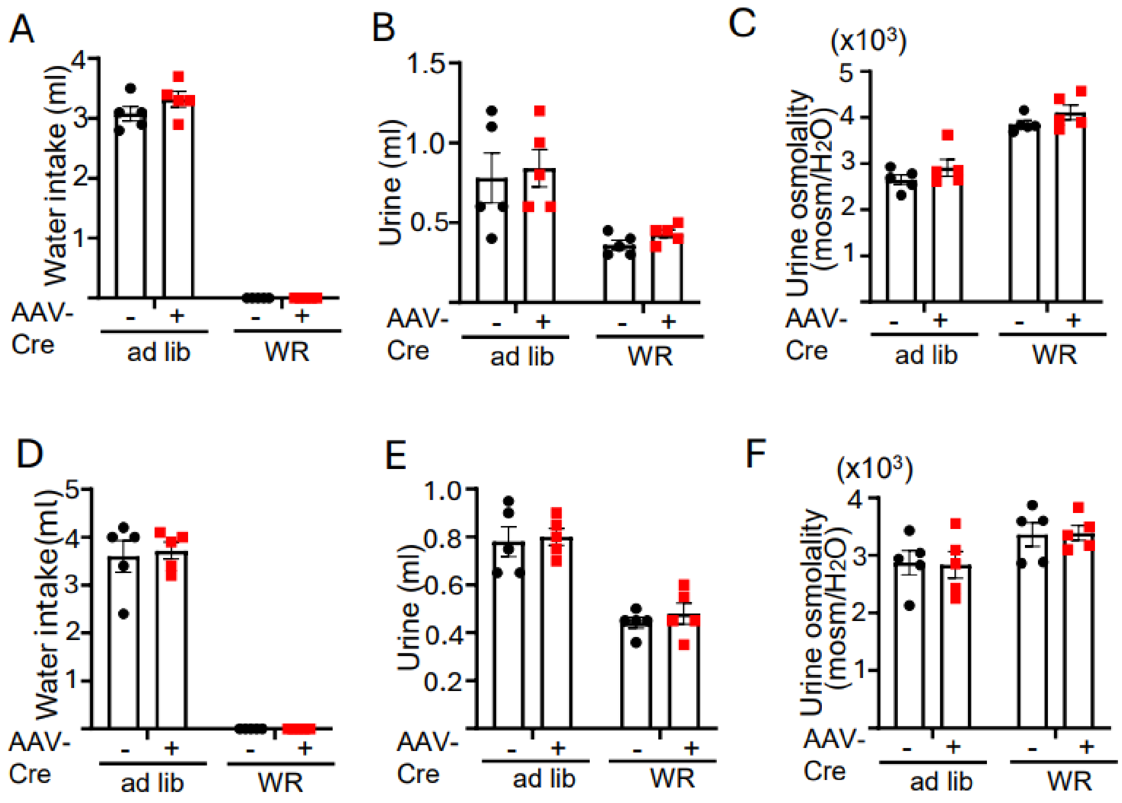

Supplementary Figure S4. Metabolic cage studies of *Osr1*$^{f/f}$*;Spak*$^{f/f}$ mice received direct injection of Cre-recombinase non-retrograde AAV virus into the PVN (A-C) or WT mice received direct injection of Cre-recombinase non-retrograde AAV virus into the OVLT (D-F). (A,D) water intake, (B,E) urine volume, and (C,F) urine osmolality before (AAV "-") and after injection (AAV "+") during *at libitum* (*ad lib*) and after water restriction (WR).

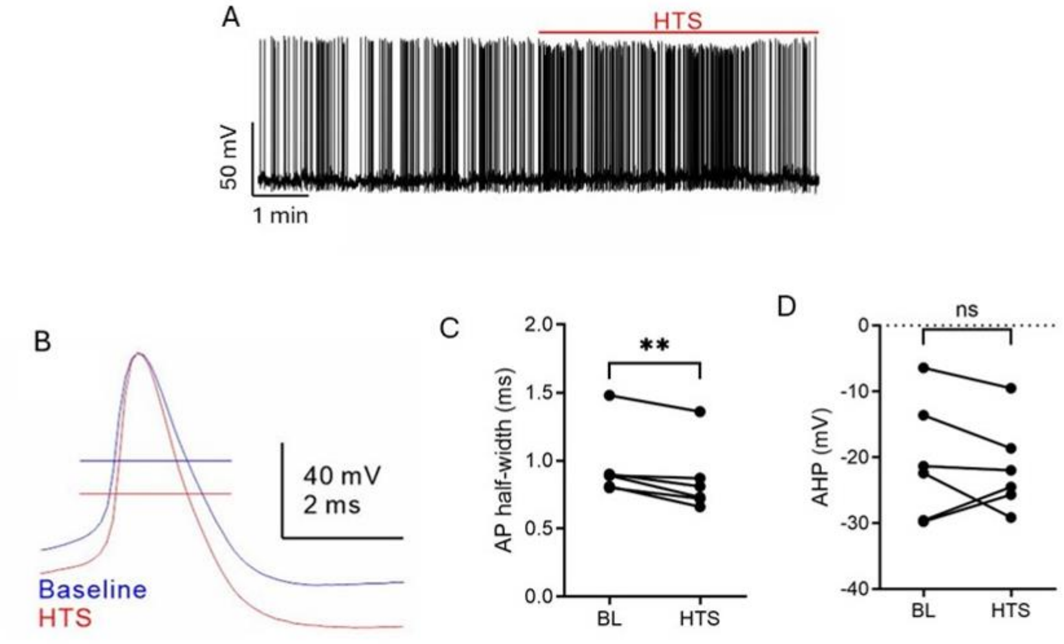

Supplementary Figure S5. Effect of HTS on action potential profile and firing. (A) Representative spontaneous action potential in OVLT neurons in isolated brain slice recorded by current clamp (see Methods) with and without HTS. Inhibition of Kv3.1 by TEA decreases the magnitude of prolonging the refractory period. (B) AP shape at baseline and after HTS. (C) AP half-width at baseline and after HTS. (D) Afterhyperpolarization potential (AHP) at baseline and after HTS. Note that HTS significantly decreases AP half-width. HTS while tends to increase AHP, it does not reach statistical significance.

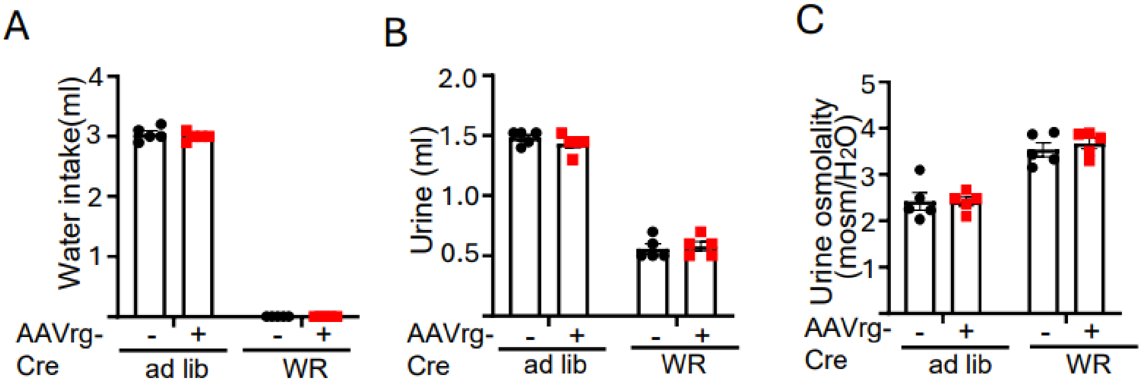

Supplementary Figure S6. Metabolic cage studies of WT mice received direct injection of retrograde AAV virus containing Cre recombinase into the PVN. (A) water intake, (B) urine volume, and (C) urine osmolality before (AAVrg-Cre "-") and after injection (AAVrg-Cre "+") during *at libitum* (*ad lib*) and after water restriction (WR).

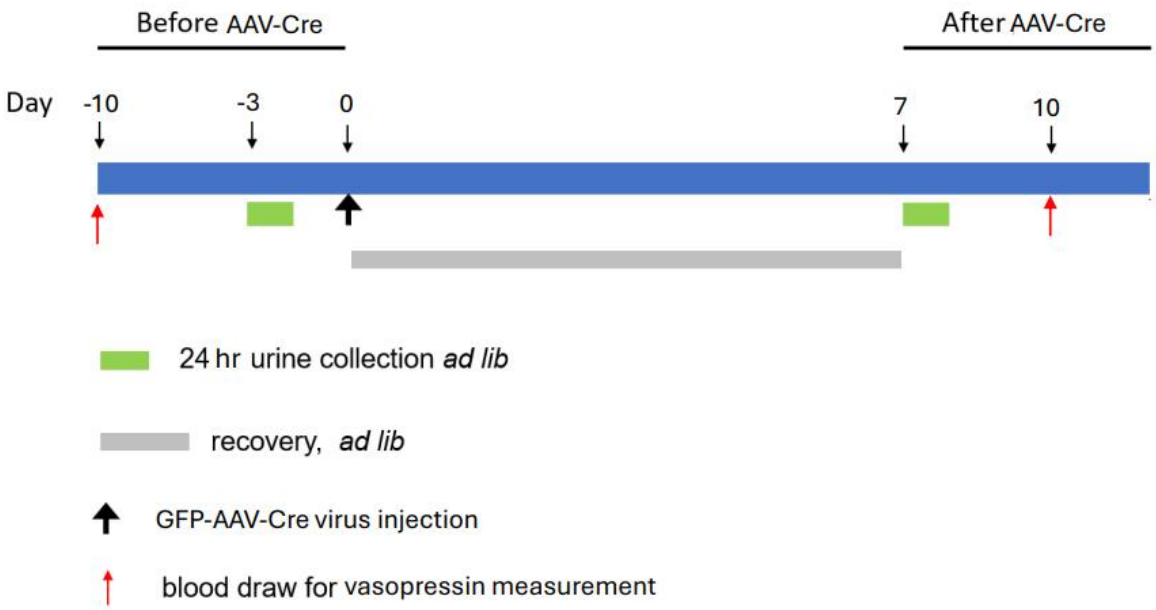

Supplementary Figure S7. Timeline for experiment with GFP-AAV-Cre virus injection into the OVLT of *CA-Osr1*$^{f/f}$ knockin mice. GFP-AAV-Cre virus were injected into OVLT at day 0. Prior to that urine collection was carried out at *ad lib* for 3 days. Urine output was monitored for ensuing 7 days after injection. Thereafter, urine collection was carried out at *ad lib* for 3 days, 24-interval. A 24-hr day starts from 12 noon to 12 noon next day. Blood was collected via retro-orbital route before and after virus injection.

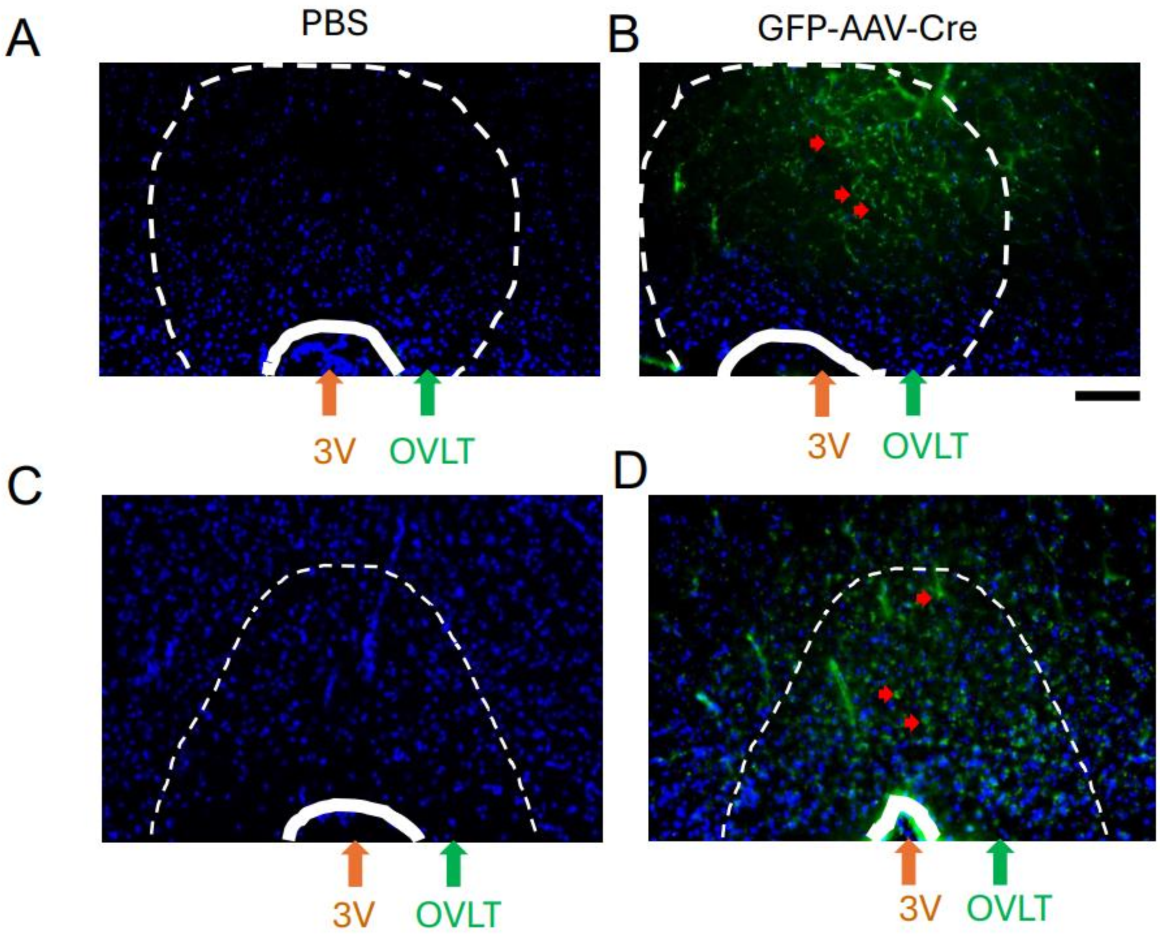

Supplementary Figure S8. GFP immunofluorescence in *Osr1*$^{f/f}$*;Spak*$^{f/f}$ (A,B) or *CA-Osr1*$^{f/f}$ knockin mice (C,D) received vehicle (PBS) or GFP-AAV-Cre virus injection into the OVLT. (A,C) as controls, PBS injections showed no green fluorescence). (B,D) GFP fluorescence in the OVLT is marked by green arrow (area between white dotted and solid line. Third ventricle (3V) is marked by orange arrow. Scale bar: 100 µm.
